# Exosomes derived from BMSCs in osteogenic differentiation promote type H blood vessel angiogenesis through miR-150-5p mediated metabolic reprogramming of endothelial cells

**DOI:** 10.1007/s00018-024-05371-4

**Published:** 2024-08-12

**Authors:** Feng Wu, Chengchao Song, Guanqi Zhen, Qin Jin, Wei Li, Xiongjie Liang, Wenbo Xu, Wenhui Guo, Yang Yang, Wei Dong, Anlong Jiang, Pengyu Kong, Jinglong Yan

**Affiliations:** 1 Department of Orthopedic Surgery, The 2nd Affiliated Hospital of Harbin Medical University, Harbin, Heilongjiang Province 150001 P. R. China; 2 College of Bioinformatics Science and Technology, Harbin Medical University, Harbin, Heilongjiang Province 150081 P. R. China; 3 School of Humanities and Social Sciences, Harbin Medical University, Harbin, Heilongjiang Province 150081 P.R. China; 4 Department of Orthopedics, Fourth Affiliated Hospital of Guangxi Medical University/Liuzhou Worker’s Hospital, Liuzhou, Guangxi Province 545000 P.R. China; 5 Department of Respiratory Diseases, Harbin Medical University Cancer Hospital, Harbin, Heilongjiang Province 150081 P.R. China; 6 Department of Gynecological Radiotherapy, Harbin Medical University Cancer Hospital, Harbin, Heilongjiang Province 150081 P. R. China

**Keywords:** Osteogenic regeneration, Bone mesenchymal stem cells, Exosome, Type H blood vessel, miRNA

## Abstract

Osteogenesis is tightly coupled with angiogenesis spatiotemporally. Previous studies have demonstrated that type H blood vessel formed by endothelial cells with high expression of CD31 and Emcn (CD31^hi^ Emcn^hi^ ECs) play a crucial role in bone regeneration. The mechanism of the molecular communication around CD31^hi^ Emcn^hi^ ECs and bone mesenchymal stem cells (BMSCs) in the osteogenic microenvironment is unclear. This study indicates that exosomes from bone mesenchymal stem cells with 7 days osteogenic differentiation (7D-BMSCs-exo) may promote CD31^hi^ Emcn^hi^ ECs angiogenesis, which was verified by tube formation assay, qRT-PCR, Western blot, immunofluorescence staining and µCT assays etc. in vitro and *in vivo.* Furthermore, by exosomal miRNA microarray and WGCNA assays, we identified downregulated miR-150-5p as the most relative hub gene coupling osteogenic differentiation and type H blood vessel angiogenesis. With bioinformatics assays, dual luciferase reporter experiments, qRT-PCR and Western blot assays, SOX2(SRY-Box Transcription Factor 2) was confirmed as a novel downstream target gene of miR-150-5p in exosomes, which might be a pivotal mechanism regulating CD31^hi^ Emcn^hi^ ECs formation. Additionally, JC-1 immunofluorescence staining, Western blot and seahorse assay results showed that the overexpression of SOX2 could shift metabolic reprogramming from oxidative phosphorylation (OXPHOS) to glycolysis to enhance the CD31^hi^ Emcn^hi^ ECs formation. The PI3k/Akt signaling pathway might play a key role in this process. In summary, BMSCs in osteogenic differentiation might secrete exosomes with low miR-150-5p expression to induce type H blood vessel formation by mediating SOX2 overexpression in ECs. These findings might reveal a molecular mechanism of osteogenesis coupled with type H blood vessel angiogenesis in the osteogenic microenvironment and provide a new therapeutic target or cell-free remedy for osteogenesis impaired diseases.

**Supplementary Information:**

The online version contains supplementary material available at 10.1007/s00018-024-05371-4.

## Introduction

Osteogenesis is very essential for skeletal system homeostasis. Dysfunction of osteogenesis can result in various bone diseases, such as delayed fracture healing, fracture nonunion, and osteoporosis [1–3]. As known, osteogenesis is tightly accompanied by angiogenesis [4]. A subtype of capillary plentiful existing in bone named type H blood vessel, which is formed by endothelial cells with high expression of CD31 and endomucin (Emcn), has been shown to highly couple with the process of osteogenesis temporally and spatially [5]. During osteogenesis, CD31^hi^ Emcn^hi^ endothelial cells (CD31^hi^ Emcn^hi^ ECs) could recruit Ostrix + osteoprogenitors to facilitate bone formation progress. Accordingly, in the osteogenesis microenvironment, osteoblasts can also secrete VEGF (vascular endothelial grow factor), SLIT3 (slit guidance ligand 3) and other cytokines to promote the proliferation, migration and differentiation of CD31^hi^ Emcn^hi^ ECs [6, 7]. This inter-coordination shows that in the osteogenic microenvironment, the communication between CD31^hi^ Emcn^hi^ ECs and osteoblasts is very tight and important. Regrettably, although bone marrow mesenchymal stem cells (BMSCs) have widely been seen as the precursor of osteoblast and used for the bone regeneration, their role in type H blood vessel formation has not been well discovered, especially their dynamic regulation and communication with CD31^hi^ Emcn^hi^ ECs in the osteogenic microenvironment.

Exosomes are extracellular vesicles with 30–200 nm in diameter with a phospholipid bilayer membrane structure. They load proteins, lipids, nucleic acids and other intercellular signaling molecules [8]. With these cargos, exosomes were demonstrated to mediate the communication between cells and material transport in the physical microenvironment, thereby mediating physiological and pathological process, even treating diseases [9]. BMSCs play a vital role in tissue repair and organ regeneration. BMSCs-derived exosomes were indicated to be the key mediators of these roles [10, 11]. BMSCs-derived exosomes also play a pivotal role in promoting healing after fractures, inhibiting the progression of osteosarcoma and treating osteoporosis [12–14]. Interestingly, these cargos in exosomes can also change during the process of BMSCs osteogenic differentiation. Therefore, we suppose that BMSCs-derived exosomes may have a potential and dynamic effect on regulating the formation of type H blood vessel in the osteogenic microenvironment.

MicroRNAs (miRNAs) are non-coding RNAs with 17–24 nt in length that mediate post-transcriptional genes by binding to the 3’ untranslated region (UTR) or open reading frame (ORF) regions of silenced receptor mRNAs [15]. As the main cargo of exosomes, miRNAs can play a regulatory role in the microenvironment. During the bone formation, a variety of exosomal miRNAs have been shown to play a regulatory role in bone regeneration and angiogenesis. BMSCs-exosomal miR-29a promotes angiogenesis by targeting VASH1 (Vasohibin 1), leading to decreased expression of VASH1 in ECs, thereby enhancing their ability of Tube formation in vitro [16]. Upregulated BMSCs-derived exosomal miR-19b can enhance the osteogenic differentiation of BMSCs through the KLF5 (Krüppel-like factor 5)/β-catenin signaling pathway to promote fracture healing. The KLF5 modulated the activation of Wnt/ β-catenin signaling pathway, which is a classical signal pathway inducing BMSCs osteogenic differentiation into osteoblast [17].

In the present study, we found that compared with BMSCs derived exosomes (BMSCs-exo), osteogenic differentiation for 7 days BMSCs derived exosomes (7D-BMSCs-exo) had greater capability to promote angiogenesis and CD31^hi^ Emcn^hi^ ECs formation in vitro. Furthermore, 7D-BMSCs-exo could enhance the bone osteogenesis by coupling the type H blood vessel angiogenesis in osteoporosis mice model. More intensely, we found that miR-150-5p expression in 7D-BMSCs-exo targeting the SOX2/PI3k/Akt pathway and improving metabolic reprogramming of the endothelial cells might be the crucial mechanism of CD31^hi^ Emcn^hi^ ECs formation during bone formation. Meanwhile, inhibition of miR-150-5p expression could promote the formation of type H blood vessel and facilitate bone osteogenesis in vivo. These results revealed a novel molecular mechanism of inducing type H blood vessel formation in the microenvironment, which provided a new treatment target in dysfunction of osteogenesis. As a cell-free treatment, 7D-BMSCs-exo could enhance angiogenesis coupled with osteogenesis to accelerate bone regeneration.

## Materials and methods

### Animals experiments

Specific pathogen-free (SPF) female C57BL/6J mice, aged 6-8weeks and weighting 16–22 g which were used to isolate BMSCs and endothelial progenitor cells (EPCs) and to establish OVX (ovariectomy)-mice models. These mice were purchased from the Animal Experiment Center of the Second Affiliated Hospital of Harbin Medical University. They were maintained under controlled conditions (22–24 °C, 55% humidity,12 h day/night rhythm) with free access to food and water during experiments. All animal care and use procedures were performed according to the Principles of Animal Care provided by the National Society for Medical Research and the Guide for the Care and Use of Laboratory Animals (Institute of Laboratory Animal Resources, NIH). All the animal experiments were approved by the ethics committee of Second Affiliated Hospital of Harbin Medical University.

The OVX-mice model was established by the ovaries surgically removed on both sides after anesthesia according to previously reported [18]. Briefly, A 50-mm incision was made in the back of the mouse, and the muscle tissue was carefully peeled off to expose the ovaries. The ovaries were removed after ligation of the fallopian tubes. Finally, the wound was sutured, and the mice were kept for another 8 weeks before they were sacrificed. For exosome experiments, the mice were randomly assigned to the BMSCs-exo and 7D-BMSCs-exo groups, which OVX-mice were injected with BMSCs derived exosomes or 7 days osteogenic differentiation BMSCs derived exosomes (100 µg/week, *n* = 5) into the tail vein for 8 weeks. For miRNA experiments, the mice were randomly assigned to the agomir NC, agomir miR-150-5p, antagomir NC and antagomir miR-150-5p group (*n* = 5). The miR-150-5p agomir, miR-150-5p antagomir and ago-mir negative control, antago-mir negative control were synthesized by RiboBio (China). Each mouse received a tail vein injection of either miR-150-5p agomir, agomir negative control, or miR-150-5p antagomir, antagomir negative control at a dose of 20 nmol in 0.2 mL saline every twice a week for 8 weeks.

### Cell culture, identification and differentiation

BMSCs were isolated from C57BL/6J mice following previous protocol [19]. The BMSCs were cultured in DMEM/F12 (1:1) medium containing 10% fetal bovine serum (FBS) and 1% double antibodies (penicillin/streptomycin mix) at 37 °C and CO2: 5%. After 72 h, unattached cells were discarded. Attached cells were considered BMSCs and passaged as passage 1 (P1) on 5 days. Then these cells were subcultured when the confluence reached 80%. P3 BMSCs were used for the next experiments. For osteogenic differentiation, BMSCs were cultured in the osteogenic induction media (Cyagen Biosciences Inc, China) for 7 days, 14 days and 21 days. For the identified of BMSCs experiments following the previous study [20], a tri-lineage-induced differentiation experiment (Osteogenic, adipogenic and chondrogenic differentiation) was performed according to the manufacturer’s instructions. Briefly, BMSCs were seeded in a 6-well plate at a density of 1 × 10^5^ cells/well in Mouse MSC Osteogenic or Adipogenic Differentiation Medium (Cyagen Biosciences Inc, China) for osteogenesis or adipogenesis induction, respectively. After 14 days, adipogenesis was assessed using oil red O staining, while osteogenesis was evaluated using alizarin red (AR) staining. For chondrogenesis, pellet culture was employed, with 1 × 10^6^ cells/tube cultured in Mouse MSC chondrogenic Differentiation Medium (Cyagen Biosciences Inc, China). After 21 days, the pellet was fixed embedded and analyzed for chondrogenesis by Alcian Blue staining.

EPCs were isolated from mice as reported previously [21, 22].Briefly, C57BL/6J mice were euthanized under sterile conditions and the femurs and tibias were obtained. Then, mononuclear cells were collected by density gradient centrifugation and transferred into dishes cultured with endothelial culture medium containing serum, growth factors, penicillin, and streptomycin (EGM-2, Lonza, Switzerland). Unattached cells were removed after 72 h, and adherent cells continued to be cultured. The cellular uptake of Dil-labeled acetylated LDL (Dil-Ac-LDL) and the binding of FITC-conjugated ulex lectin-1 (FITC-UEA-1) were examined through dual immunofluorescence staining to explore the characteristics of EPCs [23]. Briefly, cells were seeded at a density of 5 × 10^5^ cells/ml and cultured for 3 days. After twice washes with PBS at 37 °C, the cells were incubated with Dil-Ac-LDL (10 µg/ml, Maokang Biotechnology, China) for 1 h at 37 °C in the dark. Subsequently, after two additional PBS washes at 37 °C, the cells were incubated with FITC- UEA-1 (15 µg/ml, Maokang Biotechnology, China) for another 1 h at 37 °C in the dark. The cells were then analyzed for the uptake of Dil-Ac-LDL and binding of FITC-UEA-1 by fluorescence microscope.

### Flow cytometry

Flow cytometry was used to identify the phenotypic surface biomarkers of BMSCs and EPCs according to previously reported [20, 24] .Cells were washed with PBS, digested, and resuspended in 500 µl of PBS containing 1 × 106 cells. The cells were incubated with antibodies for their phenotype surface markers including CD90-PE(1: 1000; Cat#140307;Biolegend), CD73-PE(1: 1000; Cat#127205;Biolegend), CD105-APC(1: 1000; Cat#120413;Biolegend), CD34-APC(1: 1000; Cat#128611;Biolegend), CD45-FITC(1: 1000; Cat#157213;Biolegend), CD14-FITC(1: 1000; Cat#123307; Biolegend), CD34-APC(1: 1000; Cat#128611;Biolegend), CD133-APC (1: 1000; Cat#141207;Biolegend) and VEGFR2-APC (1: 1000; Cat#136405 ;Biolegend). The cells without an antibody were used as control. After an incubation for 30 min, cells were collected, and immediately analyzed by flow cytometry (CytoFLEX, Beckman, USA).

### Isolation and identification of exosomes

Before isolating exosomes, BMSCs were cultured in the Osteogenic Differentiation media for 7 days, 14 days and 21 days. BMSCs were washed with phosphate-buffered saline (PBS) and continually cultured for 48 h with exo-depleted conditioned medium (exo-depleted CM) containing DMEM/F12 (1:1) medium, 10% exo-depleted FBS and 1% double antibodies (penicillin/streptomycin mix). Exosomes were isolated from the conditioned medium through differential ultracentrifugation. In brief, the exo-depleted CM supernatant was collected and centrifuged successively at 2000 × g for 30 min at 4℃ to remove the cell debris. Then, the supernatant was collected and filtered through a 0.22 μm sterile filter. After filtration, the supernatant was centrifuged at 10,000 × g for 30 min at 4℃ to remove the subcellular components. Then the supernatant was ultracentrifuged at 100,000 × g for 70 min at 4℃ to obtain the exosomes. For purification, the pellet was re-suspended in PBS and ultracentrifuged at 100,000 × g for another 70 min at 4℃. The isolated exosomes resuspended in 100 µl of PBS were used immediately or stored at -80℃ for further experiments. The diameter distributions of exosomes were analyzed via nanoparticle tracking analysis (NTA, Beckman Coulter, USA). The morphology of exosomes was observed under a transmission electron microscope (TEM, Hitachi, Japan). Exosomes were measured for their protein content using a BCA protein assay kit (Beyotime Biotech, China). For western blotting, the exosome-specific surface markers CD63(1:1000, ab134045, Abcam), TSG101(1:1000, ab125011, Abcam) and Alix (1:1000, ab186429, Abcam) were evaluated [25, 26].

### Exosome uptake analysis

Exosomes were stained with red fluorescent dye (PKH26, Sigma, USA). After 20 min of incubation at 37℃, the exosomes were harvested and washed with PBS, and were ultracentrifuged (100,000× g, 70 min, 4℃). The EPCs nuclei were stained by DAPI dye. PKH26-labeled exosomes were cocultured with EPCs for 24 h at 37 ℃, and cellar exosomes uptake was observed under laser-scanning confocal microscope.

### In vitro cell viability

Following the manufacturer’s protocol of Cell Counting Kit-8 (Dojindo, Japan), 100 µL of cell suspension including approximately 3000 EPCs was placed in a 96-well plate and treated with BMSCs-exo (10 µg/mL), 7D-BMSCs-exo (10 µg/mL), and PBS solution. After 24 h, CCK-8 reagent (10 µL) was added, and the cells were cultivated for 3 h. Then, a spectrophotometric microplate reader was used to measure the optical density (OD) at 450 nm.

### Scratch wound assay

EPCs were seeded in the 6-well plates and cultured until reached approximately 90% confluence. A scratch of was created in the confluent cell layer by using a 200-µL pipette tip, then washed with PBS. The cells were treated with PBS, BMSCs-exo(10 µg/mL) or 7D-BMSCs-exo(10 µg/mL) in serum-free medium respectively. The plates were incubated for 24 h. Images were obtained at 0 and 24 h and analyzed using ImageJ.

### Transwell assay

EPCs were seeded on the upper chamber of 24-well Transwell plate (Corning, USA). The lower chambers were filled with BMSCs-exo (10 µg/mL), 7D-BMSCs-exo (10 µg/mL), and PBS in 20% FBS medium, respectively. The plates were incubated at 37℃. After 24 h incubating, the cells remaining on the upper side of the insert were removed using cotton swabs. The cells that migrated through the filters were fixed and stained with 0.5% crystal violet, and the images were observed under an inverted microscope. The cells were counted using ImageJ software.

### Tube formation assay

EPCs were seeded on Matrigel-coated 48-well plates (Corning, USA) and cultured in different conditional medium filled with BMSCs-exo (10 µg/mL), 7D-BMSCs-exo (10 µg/mL), and PBS, respectively. The plates were incubated at 37℃. After incubation for 6 h, tube formation was observed under an inverted microscope and the results were measured by ImageJ.

### Cell proliferation

The Cell-Light EdU Apollo 576 In Vitro Kit (Ribobio, China) was utilized to detect the ability of cell proliferation. According to the manufacturer’s protocol, EPCs were plated in 96-well plates and culturing for 24 h. The conditional medium was replaced in which added BMSCs-exo (10 µg/mL), 7D-BMSCs-exo (10 µg/mL), and PBS. The cells were imaged by fluorescence microscopy and counted automatically by Image-Pro software.

### Transfection

MiR-150-5p mimics, negative control mimics (NC- mimic), miR-150-5p inhibitor and negative control inhibitor (NC-inhibitor) were purchased from Ribobio (China). Transfections were performed by using Lipofectamine 2000 (Invitrogen, USA). Transfections were performed according to the manufacturer’s protocols.

### Western blot

Western blot procedures were performed as previously described [18, 27]. The total protein lysate was centrifuged for 15 min at 13,500 rpm at 4℃. A bicinchoninic acid (BCA) assay kit (Beyotime BioTech, China) was performed to quantify the protein concentrations. Equal amounts (20–30 µg) of protein samples were loaded and resolved on 10-15% SDS-PAGE gels and transferred to nitrocellulose membranes (Millipore, USA). The membranes were blocked in nonfat 5% milk in TBST for 1 h and then incubated with primary antibodies β-actin (1:2000,66009-1-1 g, Proteintech), CD31 (1:1000,ab28158,abcam), Emcn (1:1000,ab106100,abcam), Sox2 (1:1000,ab97959,Abcam), Hif-1α (1:1000,ab179483,abcam), HK2 (1:1000,ab209847,abcam), LDH (1:1000,ab53292,abcam), PKM2 (1:1000,ab150037,abcam), PPAR-α (1:1000,ab126285,abcam), PPAR-γ (1:1000,ab178860,abcam), MFN-1(1:1000,ab126575,abcam), MFN-2 (1:1000,ab56889,abcam), FIS-1 (1:1000,ab96764,abcam), p-PI3k (1:1000,ab182651,abcam), PI3k (1:1000,ab191606,abcam), p-AKT (1:1000,13038,CST), AKT (1:1000,9272,CST)overnight at 4 °C. The membranes were finally incubated with anti-rabbit or anti-mouse secondary antibodies for 1 h at RT after washing in TBST solution three times. The bands were detected using an ECL kit (Beyotime BioTech, China ).

### qRT-PCR

For qRT-PCR assay, we performed as previously described [28].Total RNAs were extracted from exosomes and cells by using TRIzol reagent (Invitrogen, USA) and the RNA samples were reverse transcribed to cDNA using cDNA synthesis kit. The mRNA expression was detected by SYBR Green Master Mix(Applied Biosystems, USA) and calculated by the 2^–△△CT^ method. The primer sequences were described in Additional Table 1 in this study.

### Data preparation and basic bioinformatics analyses

MicroRNA (miRNA) profiling of ten samples from BMSCs-exo and 7D-BMSCs-exo was performed sequencing using Affymetrix miRNA 4.0 Arrays. The Affymetrix data was normalized using the robust multi-array average (RMA) method as implemented in the ‘affy’ R package and removed batch effects using the ComBat method performed in the ‘sva’ R package. The differentially expressed miRNAs (DEmiRs) between BMSCs-exo and 7D-BMSCs-exo groups were identified using the ‘limma’ R package, and multiple testing corrections were performed using the Benjamini-Hochberg method (|Fold change| > 10 and *P*. adjust < 0.05). Gene Ontology (GO) and Kyoto Encyclopedia of Genes and Genomes (KEGG) enrichment analyses were analyzed using the ‘clusterProfiler’ R package. The target genes and the potential binding sites of DEmiRs were identified using the TargetScan and MiRanda databases. WGCNA (Weighted correlation network analysis) is an algorithm that describes the correlation patterns between genes in samples and finds clusters (modules) of highly related genes. Using the WGCNA algorithm (WGCNA R package) screen the miRNAs related to the osteogenic differentiation of BMSCs-exo. The optimal soft threshold was selected, and according to the correlation degree between each module and clinical pattern, the module with the highest correlation was selected as the interested clinical significance module for analysis. All reported *P*-values are two-sided, and *P*-values < 0.05 were considered statistically significant.

### Construction of weighted gene co-expression networks and identification of modules associated with clinical traits

Weighted correlation network analysis (WGCNA) was performed on miRNA expression profiles to identify the vital miRNA modules closely associated with the osteogenic differentiation progress of BMSCs. As the previous protocol [29], the analysis procedure included the following main steps: (1) constructing the coexpression network of miRNA by Pearson’s correlation analysis; (2) selecting the appropriate soft powers β to establish the scale-free topological network; (3) transforming the adjacency matrix into a topological overlap matrix (TOM); (4) performing hierarchical clustering on the coexpression network to obtain the hierarchical clustering tree; (5) using the dynamic tree cut method to identify miRNA modules with similar expression; (6) preforming Principal Component Analysis (PCA) on the miRNA in each module and the first PCA value represents the overall expression level of the module; and (7) association analysis between eigengene values and clinical traits was assessed by Pearson’s correlation. All the analysis progresses were implemented by the ‘WGCNA’ R package.

### Identification of hub miRNAs related to CD 31^hi^Emcn^hi^ endothelial cells formation

To identify the hub miRNAs associated with type “H” endothelial cells, we performed the following analysis procedure: (1) Exacting the overlapping miRNAs between the miRNAs of significant modules generated by WGCNA and DEmiRs; (2) Identifying one-step interactive neighboring genes related to CD31 and Emcn using the STRING database (www.string-db.org/); (3) The regulatory miRNAs related to the formation of type H endothelial cells were identified according to the relationships between miRNAs and their regulatory target genes; (4) The overlapping miRNAs of results above all were considered as hub miRNAs related to CD 31^hi^Emcn^hi^ endothelial cells formation.

### Luciferase assays

According to the predicted target binding site of miR-150-5p on SOX2, both wild -type and mutant pMIR-SOX2-3’UTR luciferase reporter plasmids (RiboBio, China) were obtained for luciferase reporter assay. These plasmids were co-transfected with miR-150-5p or negative control (NC) comprising of scrambled random miRNA, into 293T cells, utilizing with Lipofectamine 3000 (Invitrogen, Carlsbad, USA). The luciferase intensity was detected by the dual-luciferase reporter assay system (Promega, USA).

### Histological studies

Hematoxylin and eosin (HE) staining was utilized for histological analysis. As reported previously [30], femur were isolated from mice and fixed in paraformaldehyde solution for 24 h at 4 °C and then were decalcified in 10% EDTA (pH 7.4) solution for 21 days and embedded in paraffin. The samples were cut into 5 μm tissue sections and stained with HE solution.

### µCT analysis

Samples were scanned by µCT (90 kV, 88 µA,10 μm, µCT100 SCANCO Medical, Switzerland) from the growth plate proximally to 2 mm. For comparisons of the bone structures after different treatments, the bone parameters, including bone mineral density (BMD, mg/cm^3^), trabecular bone volume/total volume (BV/TV, %), trabecular thickness (Tb. Th, mm), trabecular separation (Tb. Sp, µm), and trabecular number (Tb. N, mm^− 1^), were calculated.

### Analysis of the metabolic profile of EPCs

Seahorse Bioscience XFe24 analyzer (Seahorse Bioscience, USA) was used to monitor the oxygen consumption rate (OCR) and extracellular acidification rate (ECAR). The assays were performed following the manufacturer’s protocol. In brief, EPCs (40,000 cells/well) were seeded in XFe24 plates for 12 h and then transfected with miR-150-5p inhibitor or miR-150-5p inhibitor NC. OCR was measured with a Cell Mito Stress Test kit under basal conditions followed by the sequential addition of 1.5 µM oligomycin, 1.5 µM FCCP and 0.5 µM rotenone & antimycin A. ECAR was measured with the Glycolysis Stress Test kit under basal conditions followed by the sequential addition of 10 mM glucose, 1 µM oligomycin, and 50 mM 2-DG. The OCR and ECAR were calculated by the Seahorse XFe24 Wave software.

### Mitochondrial membrane potential measurement

A mitochondrial membrane potential assay kit with JC-1 (Solarbio, China) was used to assess the mitochondrial membrane potential. The cells were harvested and washed with JC-1 staining buffer. The JC-1 reagent solution was added and incubated for 20 min at 37 °C in the dark. The cells were washed thrice with JC-1 staining buffer. The fluorescence of JC-1 aggregates and JC-1 monomers was analyzed using a live cell imaging system (Olympus FV10i, Japan).

#### Immunofluorescence staining assay

As our study reported [30], femur were decalcified in 10% EDTA (pH 7.4) solution for 10 days. The bone tissues were then cut into 20 μm frozen sections. Cells and sections were incubated in 0.3% Triton X-100 for 30 min and blocked with BSA solution. Then, the samples were incubated with the primary antibody at 4 °C overnight. Finally, the samples were washed three times and incubated with a fluorescein-conjugated secondary antibody for 2 h. Nuclei were stained with DAPI or Hoechst.

### Statistical analysis

All values are represented as the mean ± standard deviation (SD). GraphPad Prism 8.0 software was utilized to perform statistical analyses. All experiments were repeated at least three times. Unpaired Student’s *t* test and One-way analysis of variance (ANOVA) was used for the comparison of two or multiple groups. *P* value < 0.05 was considered statistically significant.

## Results

### Characterization of exosomes

First, we isolated and characterized BMSCs and EPCs. We found that BMSCs have a spindle-shaped morphology (Fig. S1A) and the cells had tri-lineage differentiation ability in vitro (Fig. S1B-D). EPCs could stimulate vasculogenesis, angiogenesis and osteogenesis during bone regeneration, meanwhile the crosstalk between EPCs and BMSCs could promote process of coupling of angiogenesis and osteogenesis [31, 32]. Therefore, we chose EPCs as the endothelial cells for in vitro experiments. For EPCs, the morphology at early time points (7 days) displayed irregularly spindle-shaped or rhombic, while at later time points (21 days), the EPCs exhibited the typical paving stone-like morphology (Fig. S2A). The cells could uptake both Dil- Ac-LDL and FITC-UEA-1, exhibiting characteristics of EPCs (Fig. S2B). The flow cytometry analysis showed that the positive expression of surface biomarkers in both BMSCs and EPCs was over 95%, while the negative expression was less than 5% (Fig. S1E, Fig. S2C). To elucidate the effects of different stages of BMSCs during osteogenic differentiation process on the angiogenic phenotypic changes of EPCs, we cultured the EPCs with the conditional medium of BMSCs which (BMSCs-CM) and the conditional medium of BMSCs in 7, 14, and 21days of osteogenic differentiation (7D-BMSCs-CM, 14D-BMSCs-CM,21D-BMSCs-CM). The migratory ability of endothelial cells plays a crucial role in the process of angiogenesis. So, we validated the migratory capacity of EPCs through scratch assays and Transwell assays. The results showed that compared with BMSCs-CM, only 7D-BMSCs-CM could promote EPCs migration and proliferation most significantly (Fig. S3). Therefore, we chose the BMSCs-CM and 7D-BMSCs-CM groups for the further studies.

**Fig. 1:**
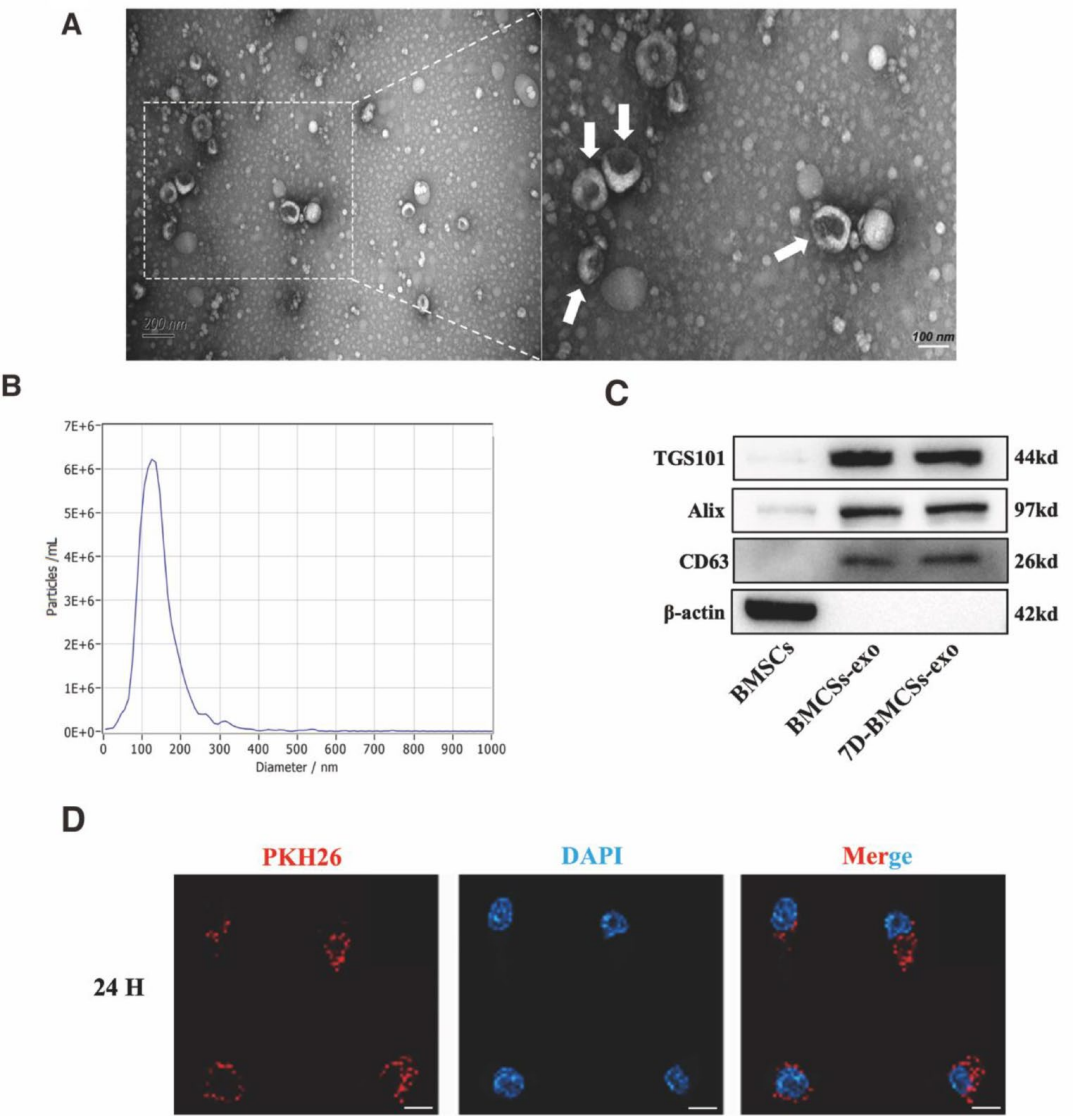
Characterization of Exosomes. (**A**) Representative transmission electron microscope image of isolated exosomes (white arrow). (**B**) Particle size distribution by nanoparticle tracking analysis (NTA). (**C**) Representative Western blotting was used to detect exosome surface markers. (**D**) Representative fluorescence microscopy image of PKH26-labeled exosomes uptake by DAPI-labeled EPCs after 24 h of coincubation. Scale bar in A = 200 nm (left), 100 nm(right), D = 20 μm

We extracted the exosomes from BMSCs-CM and 7D-BMSCs-CM by differential ultracentrifugation and identified the exosomes. Using transmission electron microscopy, we observed that the isolated exosomes were the typical spherical shape and double-layered membrane structures. The diameter of the particles is nearly 100 ~ 200 nm (Fig. 1A). The nanoparticle tracking analysis (NTA) showed that most particle sizes of the samples were approximately 100 ~ 200 nm which were in the diameter range of exosomes (Fig. 1B). Western blot analysis of the exosomes confirmed that the isolated exosomes expressed the unique exosome markers of TGS101, Alix, and CD63 and did not express β-actin (Fig. 1C). The above results indicated that the isolated exosomes meet the criteria for exosome identification. We incubated PKH26-labeled exosomes with EPCs whose nuclei were labeled by DAPI to observe intracellular localization of nuclei and exosomes. After 24 h, immunofluorescence staining indicated that the exosomes could be internalized by EPCs, potentially modulating the phenotype changes of EPCs (Fig. 1D).

### 7D-BMSCs-exo promotes ECs migration, proliferation, and CD31^hi^ Emcn^hi^ ECs formation in vitro

**Fig. 2:**
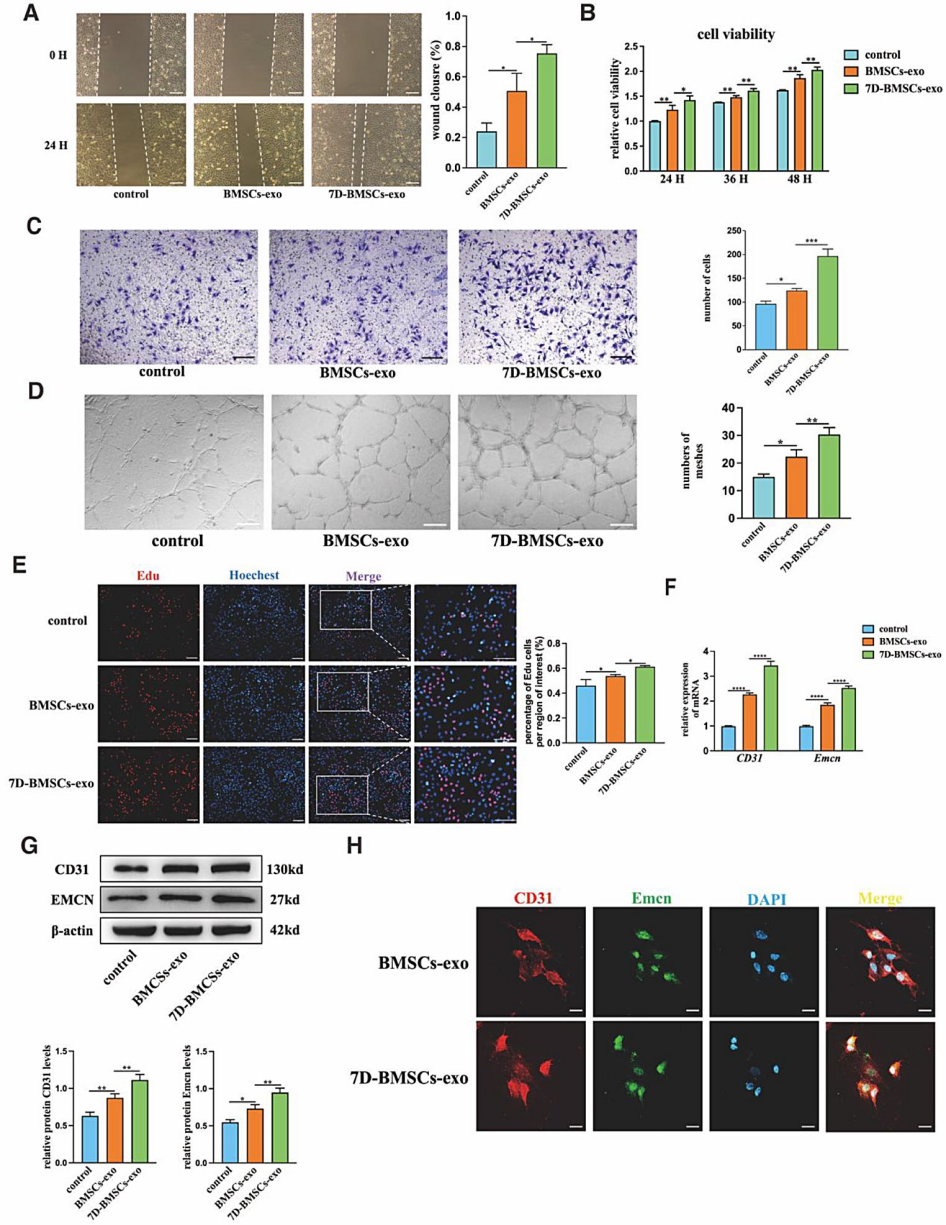
7D-BMSCs-Exo promote ECs migration, proliferation, and CD31^hi^ Emcn^hi^ ECs formation in vitro. (**A**) Representative images and statistical data showed scratch wound assays of each group at time 0 and 24 h. (**B**) Statistical data showed CCK-8 assays of EPCs co-cultured with PBS, BMSCs-exo, and 7D-BMSCs-exo at time 24 h, 36 h and 48 h. (**C**) Representative images and statistical data showed Transwell assays of each group after 24 h. (**D**) Representative images and statistical data showed tube formation assays. (**E**) Representative images and statistical data showed EdU assays of EPCs. (**F**) Statistical data showed the relative mRNA expression levels of *cd31* and *Emcm* were analyzed by qRT-PCR. (**G**) The CD31 and Emcn protein levels were analyzed by Western blot. (**H**) Representative image of immunofluorescence staining of EPCs treated with BMSCs-exo and 7D-BSMCs-exo. CD31(red), Emcn(green) and DAPI(blue). Scale bar in A = 100 μm, C = 75 μm, D = 200 μm, H = 20 μm, *P<0.05, **P<0.01, ***P<0.001, ****P<0.0001, *n* = 3

We treated EPCs with BMSCs-exo and 7D-BMSCs-exo and PBS in vitro. We found that the migration capacity of EPCs in BMSCs-exo group and 7D-BMSCs-exo group increased significantly compared with that in control group through scratch wound assay and Transwell assay. Additionally, the 7D-BMSCs-exo exhibited a more pronounced enhancement of cell migration compared to BMSCs-exo (Fig. 2A&C). Cell viability assays revealed that 7D-BMSCs-exo significantly enhanced the cell viability of EPCs (Fig. 2B). Similar results were observed in the EdU experiment. In the 7D-BMSCs-exo group, there were more EdU-positive cells, indicating a higher level of DNA synthesis and a more active proliferative state of EPCs (Fig. 2E). These findings suggested that compared to BMSCs-exo, 7D-BMSCs-exo could significantly enhance the proliferative capacity of EPCs. In the tube formation assays, we observed that there were more meshes with longer tube lengths after treating with BMSCs-exo and 7D-BMSCs-exo, especially in the 7D-BMSCs-exo group (Fig. 2D). To examine the CD31^hi^Emcn^hi^ ECs formation, the unique protein and mRNA expression levels of CD31 and Emcn in EPCs were detected by Western blot and qRT-PCR experiments. From Western blot and qRT-PCR results, we observed that 7D-BMSCs-exos could promote the expression levels of CD31 and Emcn in EPCs compared with BMSCs-exo (Fig. 2F&G). The immunofluorescence staining assay also showed that CD31 and Emcn protein expression was increased in the 7D-BMSCs-exo group than BMSCs-exo group (Fig. 2H). The results above all suggested that 7D-BMSCs-exo could enhance the EPCs migration, proliferation and promote CD31^hi^Emcn^hi^ ECs formation compared with BMSCs-exo.

### 7D-BMSCs-exo administration promotes type H vessel formation and bone regeneration in vivo

**Fig. 3:**
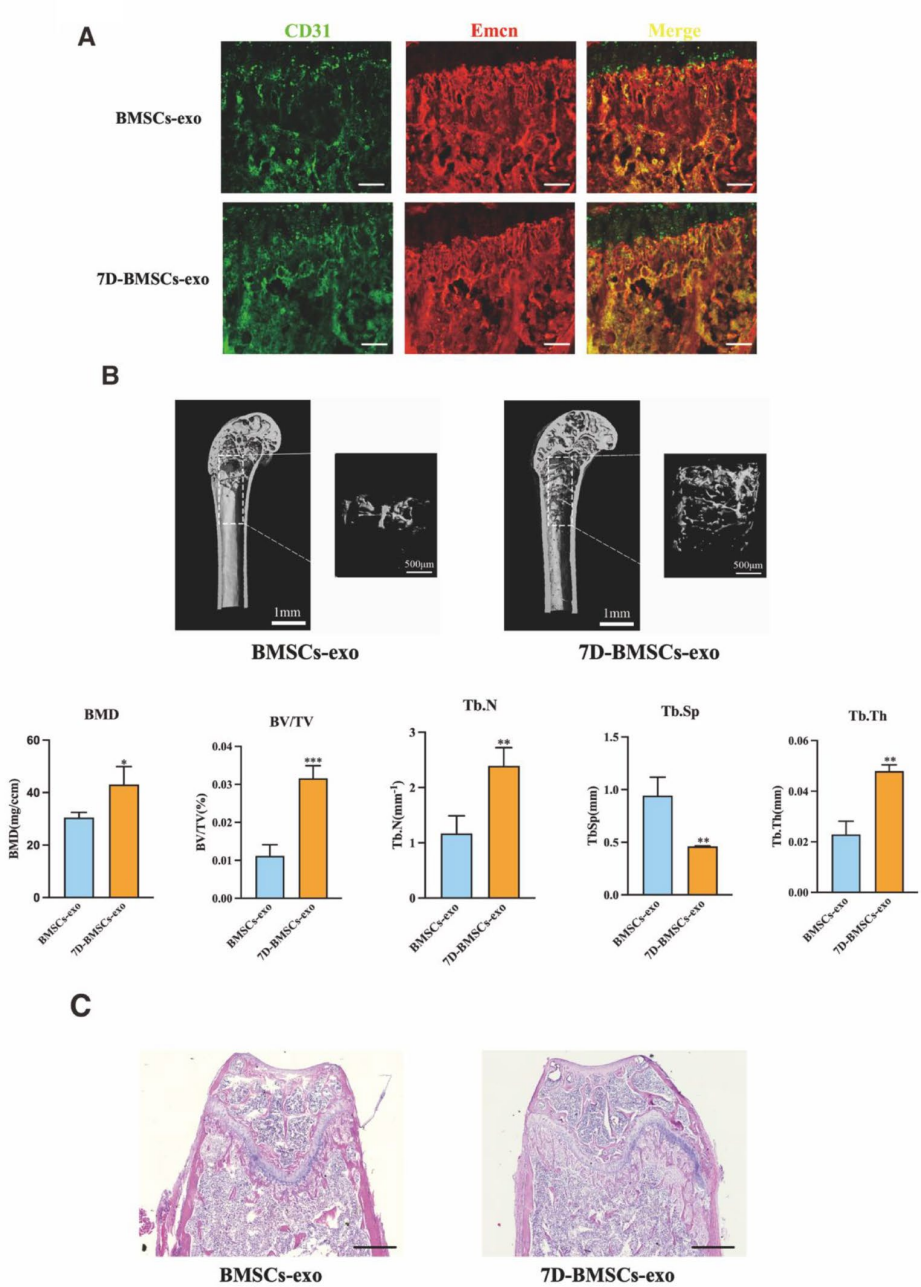
7D-BMSCs-exo administration promotes type H vessel formation and bone regeneration in vivo. (**A**) Representative images of coimmunostaining of CD31(green) and Emcn(red)in femur sections from each group. (**B**) Representative images of µCT scan and quantitative µCT analysis of trabecular bone. (**C**) Representative images of HE staining of femur of each group. Scale bar in A = 40 μm, C = 400 μm, *P<0.05, **P<0.01, ***P<0.001, ****P<0.0001, *n* = 5

Osteoporosis is a typical bone characterized by reduction in bone mass and impairment of the bone regeneration process, often highly accompanied with the type H blood vessels decrease [33]. To investigate the role of exosomes derived from BMSCs in osteogenic differentiation in promoting type H blood vessel formation coupled with the bone regeneration in vivo, we generated an OVX-induced osteoporosis mouse model in which type H blood vessel is significantly reduced. Less type H blood vessel allowed for a clearer observation of the effects of exosome administration by injecting BMSCs-exo or 7D-BMSCs-exo from the tail vein on type H blood vessel formation. We found that the quantity of type H blood vessel substantially increased after 7D-BMSCs-exo administration as indicated by CD31 and Emcn immunofluorescence staining in the metaphysis of the OVX mice femur (Fig. 3A). Furthermore, we performed a micro-CT scan to analysis bone mass in different groups. The images showed that 7D-BMSCs-exo improved OVX fragmentary bone structure. The trabecular bone volume, trabecular bone number and trabecular thickness were higher, but the trabecular separation was lower after 7D-BMSCs-exo injection (Fig. 3B). Meanwhile, the HE staining results showed that 7D-BMSCs-exo could improve trabecular structure and attenuate the bone loss in OVX osteoporosis (Fig. 3C). The results above suggested that 7D-BMSCs-exo might have a therapeutic effect on bone regeneration and could prevent osteoporosis through coupling angiogenesis and osteogenesis by inducing type H blood vessel formation.

#### Differential expresssion analysis between BMSCs-exo and 7D-BMSCs-exo and functional enrichment analysis

**Fig. 4:**
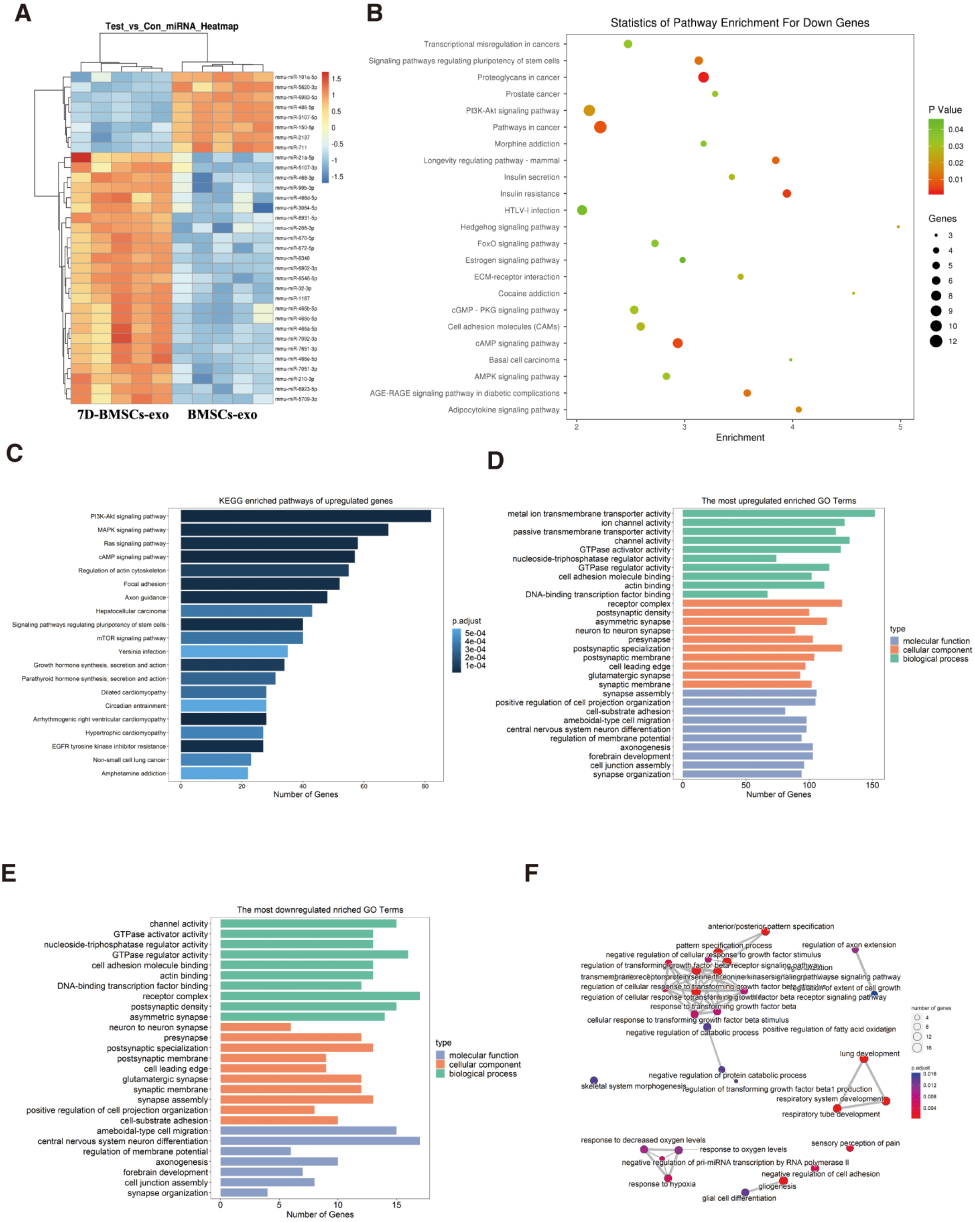
Bioinformatic functional analyses between BMSCs-exo and 7D-BMSCs-exo. (**A**) Expression heatmap of DEmiRs between 7D-BMSCs-exo and BMSCs-exo. (**B**&**C**) KEGG enrichment analyses of target genes of DEmiRs. (**D**&**E**) Top 30 GO enrichment analyses of upregulated and downregulated target genes of DEmiRs. (**F**) GO enrichment network of downregulated target genes of DEmiRs. All statistical significance satisfied adjusted *P* < 0.05

For searching for key dysregulated genes between BMSCs-exo and 7D-BMSCs-exo groups, differential expression analysis was performed. We identified 33 DEmiRs between the BMSCs-exo and 7D-BMSCs-exo, including 8 downregulated miRNAs and 25 upregulated miRNAs (Fig. 4A, Additional Table 2, FC > 10 and *P*. adjust < 0.05). Then, we predicted regulatory target genes of DEmiRs by the Miranda and TargetScan databases, and 3274 target genes were obtained by overlapping the predicted results between the two databases. To further explore the dysregulated biological characteristics and pathways affected by DEmiRs between the BMSCs-exos and 7D-BMSCs-exos groups, we performed GO and KEGG enrichment analyses for their target genes separately. According to the KEGG enrichment results, we found that both upregulated and downregulated target genes were significantly enriched in pathways such as the PI3k-Akt pathway (Fig. 4B&C), which has been reported to be related to angiogenesis [34, 35]. According to the GO enrichment results, we found that upregulated target genes were significantly enriched in GTPase activator and regulator activity, DNA-binding transcription factor binding and cell adhesion molecular binding, while downregulated target genes were significantly enriched in channel activity, asymmetric synapse, and nucleoside-triphosphatase regulator activity (Fig. 4D&E). We found that the downregulated target genes played an important role in hypoxia or response to oxygen levels (Fig. 4F). Hypoxia has been reported as a vital factor in angiogenesis and osteogenesis, especially in type H blood vessel [5, 36].

### **Identification of hub miRNAs related to the osteogenic differentiation of BMSCs-exo**

**Fig. 5:**
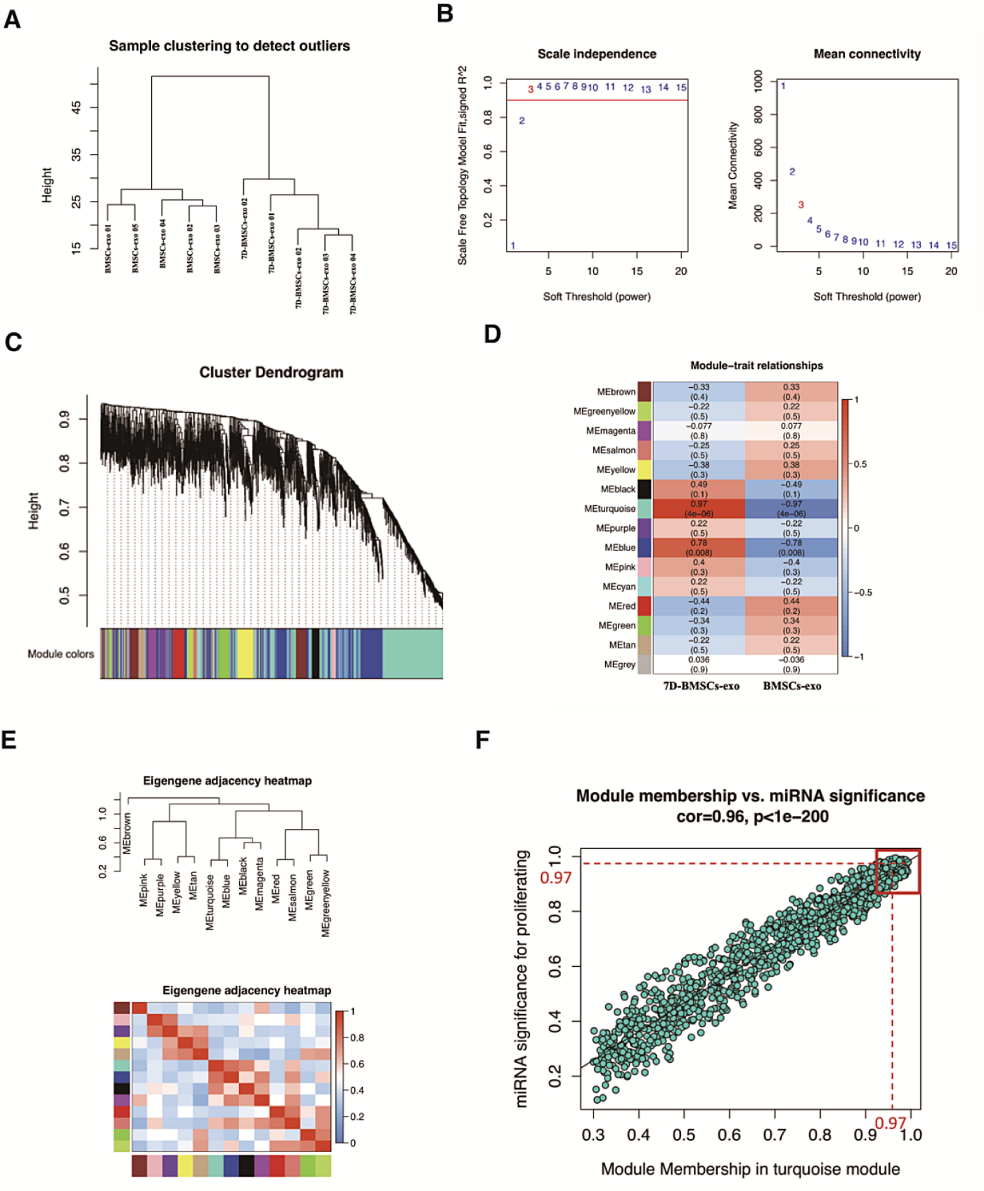
WGCNA analysis of the miRNA expression of between BMSCs-exo and 7D-BMSCs-exo. (**A**) Cluster dendrogram of samples. (**B**) Analysis of network topology for various soft-thresholding powers. (**C**) Clustering dendrograms of all DEmiRNAs, with dissimilarity based on topological overlap, together with assigned module colors. Altogether, 15 coexpression modules were constructed and displayed in different colors (**D**) Module–trait associations. Each row corresponds to a module, and each column corresponds to a trait. Each cell contains the corresponding correlation and P value. The table is color-coded by correlation according to the color legend (**E**) Eigengene dendrogram and eigengene adjacency plot. (**F**) Module and eigenmiRNA network scatterplots of MS for osteogenic differentiation progress (y-axis) vs. MM for miRNA in the turquoise module(x-axis)

To obtain exosomal miRNA closely related to the osteogenic differentiation of BMSCs, WCGNA was performed to screen the miRNAs related to the osteogenic differentiation of BMSCs-exo. We found that the samples clustered according to groups without any outlier samples in the clustering tree of the sample (Fig. 5A). All samples were deemed suitable for subsequent analysis. The power of β = 3 (scale-free R^2^ = 0.9) was selected as soft-threshold parameter to ensure that the co-expression network was a scale-free network (Fig. 5B). A total of 13 modules were identified through hierarchical clustering (Fig. 5C), in which the MEturquoise module exhibited the highest positive correlation with osteogenic differentiation progress (Fig. 5D, *r* = 0.97, *P* = 4e-06). And Fig. 5E shows the Eigengene dendrogram and eigengene adjacency plot. Therefore, the MEturquoise module was selected as the clinically significant module for further analysis. We further analyzed the correlations between miRNA and osteogenic differentiation (GS) and between miRNA and turquoise module (MM). Seventeen vital miRNAs were obtained from the turquoise module (defined as module-related miRNAs) using an MM over 0.97 and MS over 0.97 as cut-off criteria (Fig. 5F). Then, we obtained one hub miRNA (miR-150-5p) that related to the differentiation process of BMSCs-exo by intersecting DEmiRs between BMSCs-exo and 7D-BMSCs-exo with module-related miRNAs.

### The regulatory effects of miR-150-5p on type H ECs angiogenesis

**Fig. 6:**
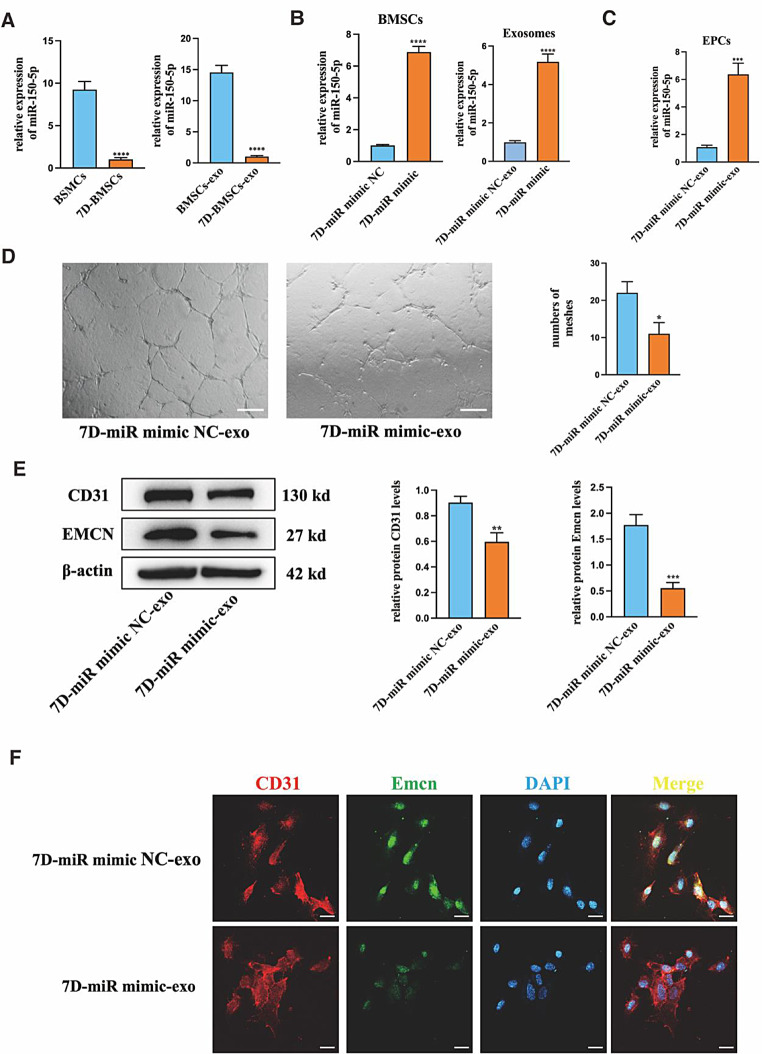
The regulatory effects of miR-150-5p on type H ECs angiogenesis. (**A**) The miR-150-5p expression in different BMSCs and exosomes. (**B**) The miR-150-5p expression in BMSCs and exosomes treated with miR-150-5p mimic and mimic NC. (**C**) The miR-150-5p expression in EPCs treated with 7D-miR mimic NC-exo and 7D-miR mimic-exo. (**D**) Representative images and statistical data showed tube formation assays of EPCs co-cultured with 7D-miR mimic NC-exo and 7D-miR mimic-exo. (**E**) Protein levels of CD31 and Emcn in EPCs treated with with 7D- miR mimic NC-exo and 7D- miR mimic-exo. (**F**) Representative image of immunofluorescence staining of EPCs treated with 7D- miR mimic NC-exo and 7D- miR mimic-exo. CD31 (red), Emcn (green) and DAPI (blue). Scale bar in D = 200 μm, F = 20 μm. *P<0.05, **P<0.01, ***P<0.001, ****P<0.0001, *n* = 3

To determine whether miR-150-5p plays a vital role in CD31^hi^Emcn^hi^ ECs angiogenesis, the expression of miR-150-5p in BMSCs and exosomes was first detected. The qRT-PCR results showed the expression of miR-150-5p decreased in both cells and exosomes when BMSCs were differentiated in the osteogenic medium for 7 days (Fig. 6A). Then, the miR-150-5p mimic or mimic negative control (mimic NC) was transfected into BMSCs undergoing 7 days of osteogenic differentiation, and their exosomes were extracted as 7D-miR mimic-exo and 7D-mimic NC-exo respectively. qRT-PCR results demonstrated that with the overexpression of miR-150-5p in BMSCs, its expression in exosomes was also increased (Fig. 6B). In addition, the content of miR-150-5p was enhanced in EPCs with the co-culture of 7D-miR mimic-exo (Fig. 6C). The tube formation assays showed that the number of meshes, and the length of tube structure were all decreased, and Western blot results also showed that the expression of CD31 and Emcn proteins was reduced in the 7D-miR mimic-exo group, when compared to the 7D-miR mimic NC-exo group (Fig. 6D&E). Immunofluorescence staining showed a similar trend that overexpression of miR-150-5p in BMSCs derived exosomes suppressed the CD31^hi^ Emcn^hi^ formation (Fig. 6F). These results illustrated that the downregulated miR-150-5p in exosomes might be the key factor in CD31^hi^Emcn^hi^ ECs angiogenesis.

### MiR-150-5p promoted CD31^hi^Emcn^hi^ ECs formation by targeting *Sox2*

**Fig. 7:**
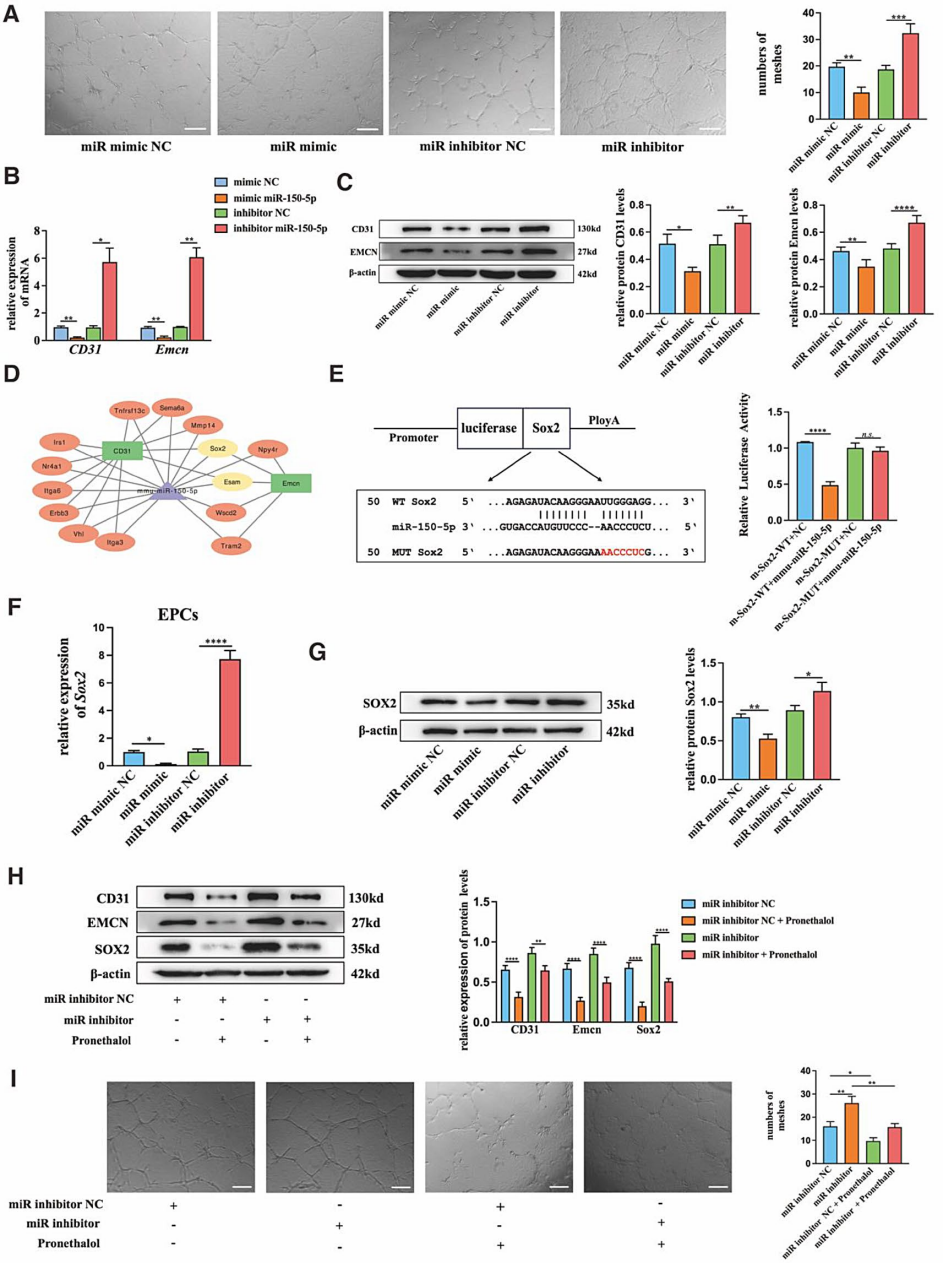
MiR-150-5p promoted CD31^hi^Emcn^hi^ ECs formation by targeting *Sox2.* (**A**) Representaive images and statistical data showed tube formation assays of EPCs transfected with miR-150-5p mimic/inhibitor. (**B**) The relative mRNA expression of *cd31* and *emcn* in each group. (**C**) Western blot analysis and quantification of CD31 and Emcn protein in each group. (**D**) miRNA-target gene regulatory network. (**E**) Predicted binding sites or mutations of miR-150-5p in the *Sox2* mRNA 3’UTR and dual-luciferase reporter assay. (**F**) The relative mRNA expression of *Sox2* in each group. (**G**) Western blot analysis and quantification of SOX2 protein in each group. (**H**) Western blot analysis and quantification of CD31, Emcn and SOX2 protein in EPCs treated with miR-150-5p inhibitor, inhibitor NC and pronethalol. (**I**) Representative images and statistical data showed tube formation assays of EPCs treated with miR-150-5p inhibitor, inhibitor NC and pronethalol. Scale bar in A, I = 200 μm. *n.s. P*>0.05, *P<0.05, **P<0.01, ***P<0.001, ****P<0.0001, *n* = 3

To demonstrate the potential mechanism by which miR-150-5p regulates CD31^hi^Emcn^hi^ ECs formation, miR-150-5p inhibitor and inhibitor negative control (inhibitor NC) were transfected into EPCs. Compared to the miR inhibitor NC group, the number of meshes was more and the tube length was longer in miR inhibitor group in tube formation assays (Fig. 7A). qRT-PCR and Western blot results showed that the relative mRNA or protein levels of CD31 and Emcn were all increased in miR inhibitor group (Fig. 7B&C). Then, we obtained 2167 type H blood vessel-related genes by extracting the one-step interactive genes related to CD31 and Emcn in the protein-protein interaction network. In addition, 77 target genes of mmu-miR-150-5p were obtained from the DEmiR-target gene relationships, of which 14 target genes were related to type H blood vessel angiogenesis. Thus, we constructed a miRNA-gene network to analyze the relationship between miR-150-5p and type H blood vessel angiogenesis. As shown, we found that 9 target genes of miR-150-5p only had a direct interaction with CD31, and 3 target genes of miR-150-5p only had direct interaction with Emcn. While *Sox2* and *Esam* had direct interactions with CD31 and Emcn, implying the important role of these two genes in type H blood vessel angiogenesis (Fig. 7D). Based on the online tools (TargetScan and Miranda), SOX2 (SRY-Box Transcription Factor 2) was thought to be a potential target gene because it has a binding site for miR-150-5p within its 3’-UTR. The dual-luciferase reporter assay revealed that the luciferase activity of Sox2-WT 293T cells transfected with plasmid containing the wild type 3’-UTR of Sox2 (pmir-m-Sox2-WT) was reduced by the transfection with the miR-150-5p mimic. However, transfection of the miR-150-5p mimic had no effect on the luciferase activity of Sox2-MUT 293T cells transfected with plasmid-labeled mutated Sox2 3’-UTR (pmir-m-Sox2-MUT) (Fig. 7E). qRT-PCR results showed that the mRNA expression of *Sox2* was enhanced by the transfection of miR-150-5p inhibitors in EPCs, and the protein level of SOX2 also increased with the inhibitor treatment (Fig. 7F&G). All these results supposed that *Sox2* might be the target gene of miR-150-5p. Then, pronethalol was used to inhibit the expression of SOX2 [37]. Western blot results revealed that pronethalol could inhibit the increased protein level of SOX2 caused by the miR-150-5p inhibitor. The tube formation assay showed that the number of meshes and length of tube structure were both decreased by the addition of pronethalol. The protein expressions of CD31 and Emcn were all suppressed by the SOX2 inhibition of pronethalol (Fig. 6H&I). According to the results above, the miR-150-5p targeting *Sox2* might be the pivotal molecular basis by which exosomes derived from 7D-BMSCs mediate CD31^hi^ Emcn^hi^ ECs formation.

## MiR-150-5p improves CD31^hi^ Emcn^hi^ECs formation by shifting metabolic reprogramming via targeting the SOX2/PI3k/Akt axis

**Fig. 8:**
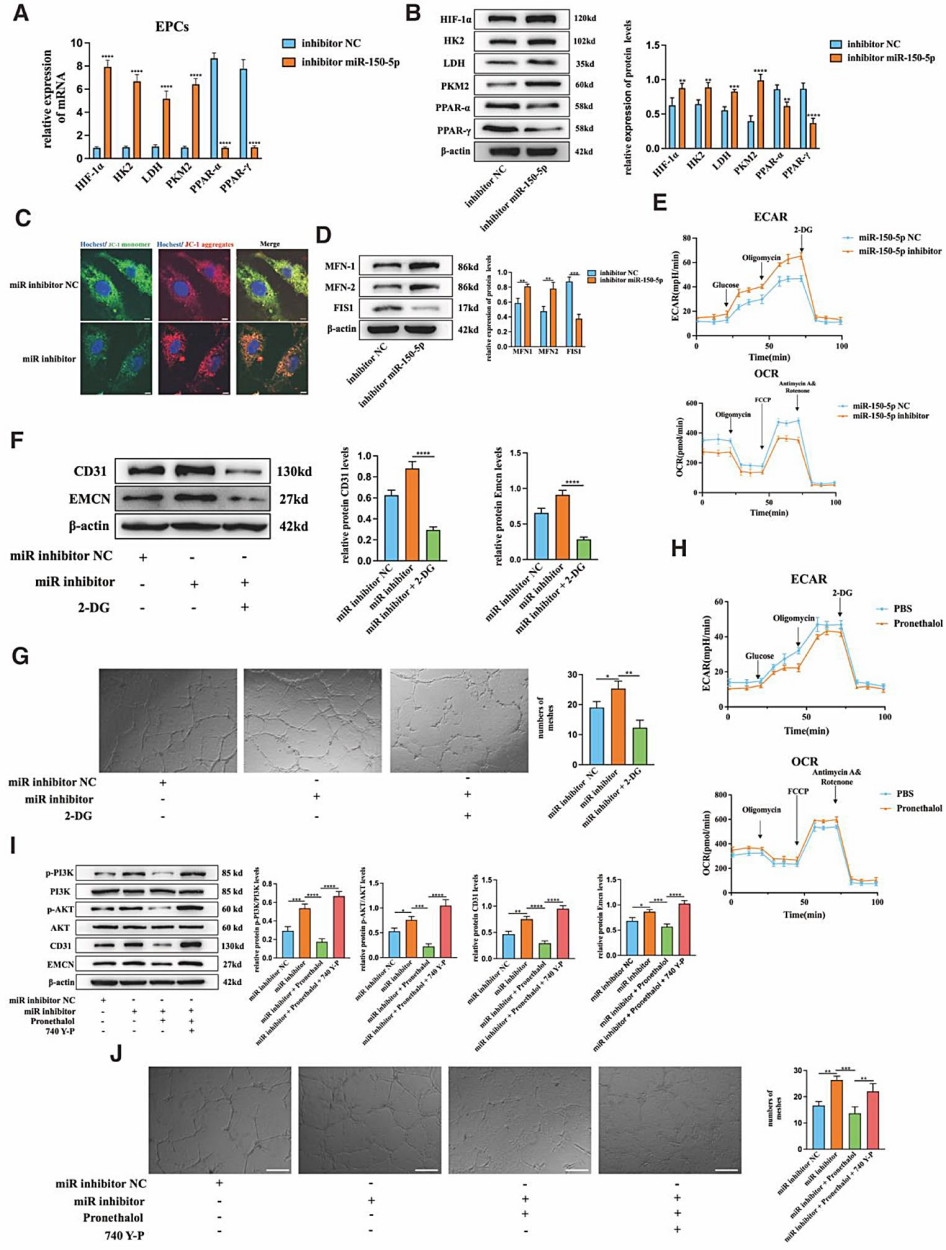
MiR-150-5p improves CD31^hi^Emcn^hi^ ECs formation by shifting metabolic reprogramming via targeting the SOX2/PI3k/Akt axis. (**A**) The relative mRNA expression levels of *hif-1α*,* hk2*,* ldh*,* pkm2*,* ppar-α* and *ppar-γ* in each group. (**B**) Western blot analysis and quantification of HIF-1α, HK2, LDH, PKM2, PPAR-α, PPAR-γ protein in each group. (**C**) Representative images of JC-1 immunofluorescence assays. Blue: Hoechst, Green: JC-1 monomer, Red: JC-1 aggregates. (**D**) Western blot analysis and quantification of MFN-1, MFN-2 and FIS-1 protein in each group. (**E**) Cellular oxygen consumption rate (OCR) and extracellular acidification rate (ECAR) levels at the indicated times of treatment with inhibitor NC and miR-150-5p inhibitor. (**F**) Western blot analysis and quantification of CD31 and Emcn protein in each group. (**G**) Representative images and statistical data showed tube formation assays of EPCs treated with inhibitor NC, miR-150-5p inhibitor and 2-DG. (**H**) Cellular oxygen consumption rate (OCR) and extracellular acidification rate (ECAR) levels at the indicated times of treatment with Pronethalol. (**I**) Western blot analysis and quantification of PI3k/p-PI3k, Akt/p-Akt, CD31 and Emcn protein in each group. (**J**) Representative images and statistical data of tube formation assays of EPCs treated with inhibitor NC, miR-150-5p inhibitor ,2-DG and pronethalol. Scale bar in C = 5 μm, F&I = 200 μm. *P<0.05, **P<0.01, ***P<0.001, ****P<0.0001, *n* = 3

It has been widely accepted that metabolic reprogramming participates in the phenotypic changes and functions of cells [38, 39]. The downregulated target gene also significantly associated with hypoxia or response to oxygen levels which could affect metabolic reprogramming (Fig. 4F). Therefore, we assessed the metabolic status of EPCs after the transfection with miR-150-5p inhibitor NC and miR-150-5p inhibitor. qRT-PCR and Western blot results showed that compared to inhibitor NC treatment, the miR-150-5p inhibitor increased the expression levels of mRNA and protein levels of Hif-1α, HK2, LDH and PKM2, and decreased the expression levels of PPARα and PPARγ (Fig. 8A&B). JC-1 staining indicated that the red aggregation of JC-1 in the miR-150-5p inhibitor group was less, which meant that the membrane potential of mitochondria was lower (Fig. 8C). Meanwhile, the fusion proteins (MFN1 and MFN2) and fission protein (FIS1) which reflect a dynamic balance of mitochondrial fusion and fission were all detected by Western blot. The results demonstrated that with the effect of the miR-150-5p inhibitor, the protein levels of MFN1 and MFN2 were increased and the protein level of FIS1 was decreased (Fig. 8D). This result indicated that the mitochondrial fusion cycles were accelerated by treatment with the miR-150-5p inhibitor while fission cycles were restrained. Seahorse assay was performed to determine the carbohydrate metabolism pattern. The OCR showed decreased oxygen consumption and the ECAR showed an increased extracellular acidification rate in the miR-150-5p inhibitor treatment group (Fig. 8E). Then, 2-DG (2-deoxy-D-arabinohexose) was used to suppress the glycolysis of EPCs. The addition of 2-DG reversed the increased expression of CD31 and Emcn proteins and inhibited the tube formation in EPCs affected by the miR-150-5p inhibitor (Fig. 7F&G). These results indicated that miR-150-5p might facilitate the metabolic shift from OXPHOS (oxidative phosphorylation) to glycolysis in EPCs, and the metabolic reprogramming might have great effects on the function played by miR-150-5p. Sox2, the target of miR-150-5p, was reported to mediate the metabolic reprogramming and angiogenesis activity [40, 41]. The Seahorse assay indicated that pronethalol, which is the inhibitor of Sox2 could increase the oxygen consumption and decrease the extracellular acidification rate in EPCs (Fig. 8H). These results demonstrated that Sox2 played a key role in the metabolic reprogramming and angiogenesis activities of CD31^hi^ Emcn^hi^ ECs formation.

The PI3k/Akt signaling pathway has great influence in the formation of CD31^hi^ Emcn^hi^ ECs [42]. According to bioinformatic analysis results, the PI3k/Akt signaling pathway was significantly enriched in downregulated target genes by KEGG analysis (Fig. 4B). Therefore, we focused on whether miR-150-5p/Sox2 could influence through PI3K/Akt signaling pathway inducing CD31^hi^ Emcn^hi^ ECs formation. Western blot results revealed that the phosphorylation of PI3k and Akt was promoted by the miR-150-5p inhibitor and weakened by the addition of Pronethalol. PI3K agonist 740 Y-P could activate the phosphorylation of PI3K and Akt. 740 Y-P could all increase the descending trend of CD31 and Emcn caused by SOX2 inhibition. In addition, 740 Y-P could improve the number and length of tube structures in pronethalol-treated EPCs. These results indicated that the function performed by SOX2 might depend on the PI3K/Akt signaling pathway (Fig. 8I&J).

### Inhibiting the expression of of miR-150-5p promotes type H vessel formation and osteogenesis in vivo

**Fig. 9:**
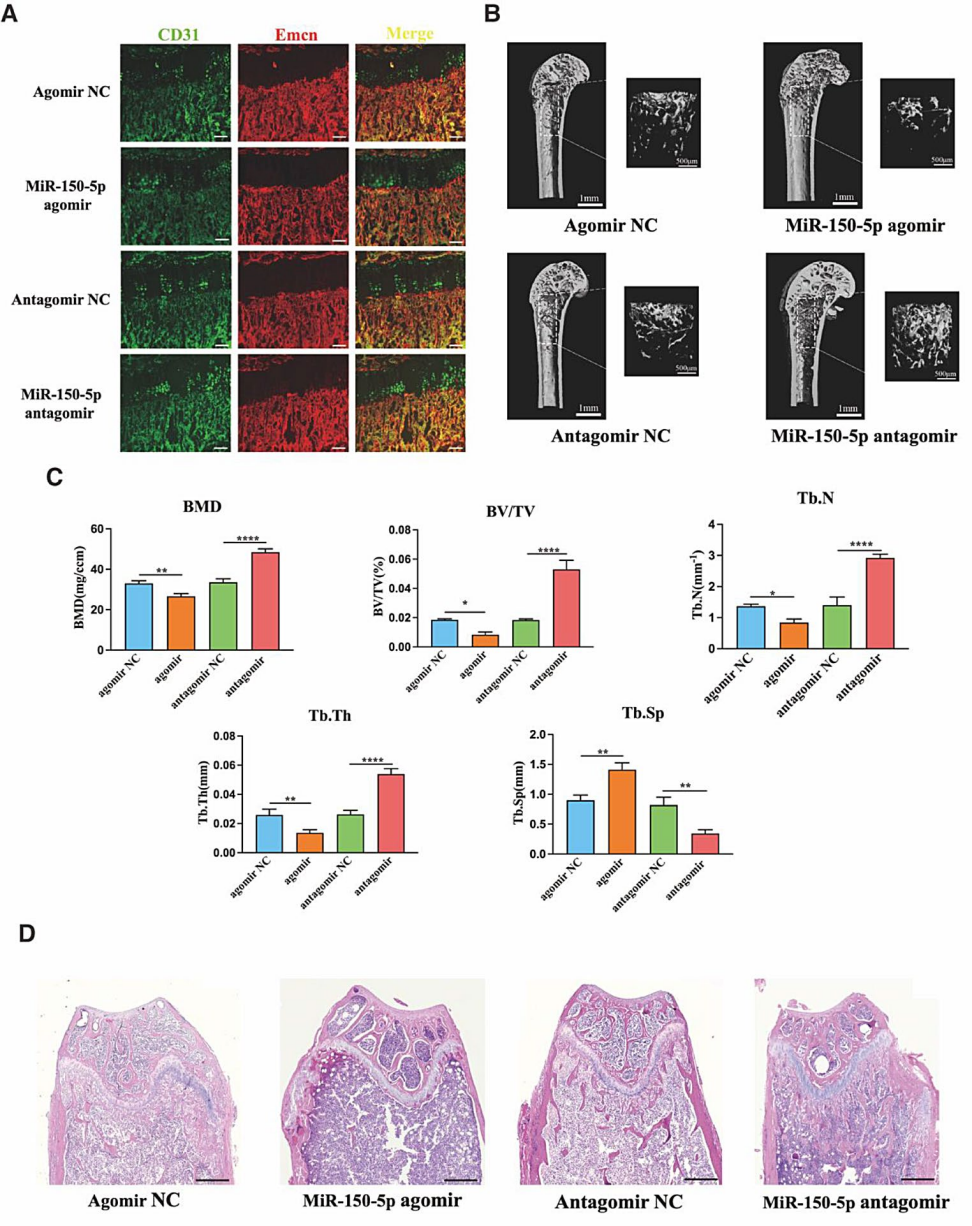
Inhibiting the expression of of miR-150-5p promotes type H vessel formation and osteogenesis in vivo. (**A**) Representative images of coimmunostaining of CD31(green) and Emcn (red) in femur sections from each group. (**B**&**C**) Representative images of µCT scan and quantitative µCT analysis of trabecular bone. (**C**) Representative images of HE staining of femur of each group. Scale bar in A = 40 μm, C = 400 μm, *P<0.05, **P<0.01, ***P<0.001, ****P<0.0001, *n* = 5

To explore the impact of miR-150-5p on type H blood vessel formation coupled with the bone regeneration in vivo, the OVX-induced osteoporosis mice were injected the sustained activator of miR-150-5p, agomir miR-150-5p, as well as its inhibitor, antagomir miR-150-5p and their respective negative control reagents though the tail vein. CD31 and Emcn immunofluorescence staining near the metaphysis of the OVX mice femur showed the antagomir miR-150-5p could elevate the type H blood vessel formation, while the agomir miR-150-5p inhibited the type H blood vessel formation (Fig. 9A). This indicated that inhibiting the expression of miR-150-5p might enhance the formation of type H blood vessels *in vivo.* The micro-CT images indicated that antagomir miR-150-5p could improve the OVX fragmentary bone structure, increase the bone mass and trabecular bone formation. The bone mineral density, trabecular bone volume and number and trabecular thickness were higher, but the trabecular separation was lower after antagomir miR-150-5p administration. Whereas in the agomir miR-150-5p group, the results above reversed significantly. The administration of agomir miR-150-5p would reduce the bone regeneration and enhance the bone loss in osteoporosis (Fig. 9B&C). Additionally, the HE staining results revealed that antagomir miR-150-5p could improve trabecular structure and attenuate the bone loss in OVX osteoporosis (Fig. 9D). These results demonstrated that reduced expression of miR-150-5p might enhance bone regeneration due to inducing the couple of type H blood vessel angiogenesis and osteogenesis in vivo.

## Discussion

The skeleton plays a vital role in supporting body posture and movement, protecting fragile organs, and storing the mineral molecules [43]. The balance of skeletal homeostasis is the cornerstone of bone health. Maintaining the skeletal homeostasis relies on abundant critical cell and molecular factors in the bone niche microenvironment [44]. In the process of bone formation, bone marrow mesenchymal stem cells generate osteoblasts, and their progenitors are essential for bone homeostasis [45]. Meanwhile, the ancillary cell types are present in bone formation. For example, a subset of CD31^hi^ Emcn^hi^ vascular endothelial cells (CD31^hi^ Emcn^hi^ ECs) mediates the coupling of angiogenesis and osteogenesis in the microenvironment of bone regeneration by promoting the maturation of perivascular osteoprogenitor cells [5]. During the osteogenic differentiation process, the molecular mechanism of the interaction between BMSCs and CD31^hi^ Emcn^hi^ ECs in the microenvironment is unclear.

Exosomes play pivotal roles in intercellular communication in the microenvironment [46]. In this study, we obtained BMSCs derived exosomes with or without osteogenic differentiation for 7 days. We found that the exosomes could promote the migration and proliferation of ECs and the differentiation of CD31^hi^ Emcn^hi^ ECs in vitro and in vivo. Among these results, the osteogenic BMSCs derived exosomes might regulate the couple of osteogenesis and angiogenesis. Similar results were also revealed on the previous studies that BMSC-derived exosomes could promote angiogenesis and osteogenesis [16]. The couple of osteogenesis and angiogenesis shows extremely rigid temporal and spatial coherence [47].Furthermore, we found that 7D-BMSCs-exo had a stronger ability to promote type H blood vessel formation than BMSCs-exo. This indicated that the osteogenic differentiating BMSCs might accelerate blood vessel formation, especially type H blood vessel through secreting different exosomes. We also found that the exosomes derived from BMSCs during different osteogenic phases possess varying abilities to promote angiogenesis. At 7 days, BMSCs are in the early stage of osteogenic differentiation into osteoblasts, and the 7D-BMSCs-exo have most mighty angiogenesis ability. The period of 7 days is also the phase hematoma formation and flourishing period of blood vessel formation after bone fracture [48]. These results above imply that the exosomes in the osteogenic microenvironment might be the key substance of maintaining the tightly spatiotemporal couple between the osteogenesis and angiogenesis.

MiRNAs are the main cargo that regulate the molecular function of exosomes [49]. Plentiful of miRNAs mediate angiogenesis in bone formation and regeneration [50, 51]. To clarify the mechanism by which BMSCs promote type H blood vessel formation, we screened the DEmiRNAs in the two groups of exosomes by miRNA microarray analysis and 33 DEmiRs were identified. Furthermore, according to GO enrichment analysis, we found that hypoxia or response to oxygen levels functions were correlated with differentially expressed miRNAs between BMSCs-exo and 7D-BMSCs-exo groups. The hypoxia and hypoxia-inducible factor-1 alpha (HIF-1α) pathway is tightly correlated with angiogenesis and osteogenesis [36, 52, 53]. Activation of the hypoxia signaling pathway in ECs could increase type H blood vessel [5]. Previous study demonstrated that the miR-497 ~ 195 cluster regulated CD31^hi^ Emcn^hi^ vessel angiogenesis by targeting HIF-1α activity [54]. Among the results of the KEGG enrichment analysis, PI3k/Akt signaling pathway was enriched and it was reported to modulate angiogenesis in the past studies [34, 35, 55]. These results indicate the 7D-BMSCs-exo might promote type H vessels formation by the activation of hypoxia and PI3K-Akt signaling pathways. We also utilized the WGCNA algorithm to cluster the differentially expressed miRNAs for further screening the modules related to the osteogenic differentiation process of BMSCs. WGCNA is an ingenious bioinformatic algorithm that can obtain highly biologically significant co-expression network modules to screen out the genes related to clinical traits [29]. We screened out the exosomal miRNA module that was most tightly associated with the osteogenic differentiation of BMSCs. The 17 miRNAs in the module could rigidly reflect the osteogenic differentiation ability of BMSCs. Exosomal miRNAs are widely utilized as a potential biomarker for early diagnosis and prognosis of disease [56, 57]. Among the module miRNAs, the exosomal miR-150-5p which was most associated with osteogenic differentiation of BMSCs might be a potent biomarker for prognosis of bone regeneration damage disease in the early stage.

We identified the exsomal miR-150-5p might be the potential hub miRNA for the CD31^hi^ Emcn^hi^ ECs formation through regulating metabolic reprogramming and the PI3k/Akt signaling pathway by targeting *Sox2* during the BMSCs osteogenic differentiation. MiR-150-5p has been reported to modulate angiogenesis progress in various diseases by targeting *vegfa*, *mmp9*, and *mmp14* [58–60].SOX2 (SRY-Box Transcription Factor 2) is a member of the Sry-related HMG box (SOX) transcription factor family [52]. It has been proven that SOX2 could regulate tumorigenesis and metastasis, stemness and induction of neural stem cells, and developmental processes [61–63]. The roles of SOX2 in enhancing angiogenesis in cancers and the wound healing process have also been reported [64, 65]. In our study, we found the inhibitor of miR-150-5p could elevate CD31^hi^ Emcn^hi^ ECs formation accompanying the overexpression of SOX2 and altering oxidative phosphorylation into glycolysis in EPCs. This metabolic reprogramming mechanism induced by SOX2 could modulate sorts of malignant tumor cells to enhance aggressive phenotypes, drug resistance and metastatic ability, such as in melanoma and prostate cancer [40, 66]. Further studies suggested that SOX2 could enhance CD31^hi^ Emcn^hi^ ECs formation correlated with the PI3k/Akt pathway. Chen et al. found that PI3k/Akt/SOX2 axis might regulate the stemness of diffuse large B cell lymphoma in non-Hodgkin lymphoma [67]. Li et al. reported that pharmacological intervention will activate the PI3k/Akt signaling pathway by increasing SOX2 levels [68]. In our research, we found that the overexpression of SOX2 in EPCs accompanied the activation of the PI3k/Akt signaling pathway. This might be another potential molecular mechanism by which SOX2 mediating CD31^hi^ Emcn^hi^ ECs formation.

To summarize, exosomes derived from 7 days osteogenic differentiation BMSCs could increase endothelial cells proliferation, migration and CD31^hi^ Emcn^hi^ ECs formation. Furthermore, we found that miR-150-5p is highly associated with the communication between BMSCs and CD31^hi^ Emcn^hi^ ECs in the osteogenic microenvironment. SOX2 might be the target gene of miR-150-5p in the regulation, and the overexpression of SOX2 could induce CD31^hi^ Emcn^hi^ ECs formation by regulating oxidative phosphorylation and the PI3k/Akt signaling pathway. From these results, we first suggest the hypothesis that osteogenic BMSCs derived exosomes might be the vital cell-cell communication junctions between osteogenesis and CD31^hi^ Eman^hi^ blood vessel formation. MiR-150-5p/Sox2 might be the molecular mechanism of this phenomenon.

## Conclusion

Overall, our study found that exosomes derived from BMSCs in 7 days osteogenic differentiation promoted endothelial cells proliferation, migration and CD31^hi^ Emcn^hi^ endothelial cells formation compared with BMSCs-exo. MiR-150-5p targets SOX2 and it might be the essential miRNA mediating CD31^hi^ Emcn^hi^ ECs formation by regulating metabolism and PI3k/Akt in the osteogenic microenvironment. These results suggested a novel mechanism of type H blood vessel formation in osteogenesis that could be utilized as a potential therapeutic target and a cell-free therapy in bone regeneration damage diseases.

### Electronic supplementary material

Below is the link to the electronic supplementary material.

Supplementary Material 1

Supplementary Material 2

Supplementary Material 3

Supplementary Material 4

Supplementary Material 5

## Data Availability

The data that support the findings of this study are available from the corresponding author upon reasonable request.

## Abbreviations

2-DG: 2-Deoxy-D-arabino-hexose
7D-BMSCs-exo: Osteogenic differentiation for 7 days bone marrow mesenchymal stem cells exosomes
BCA: Bicinchoninic acid assay
BMSCs-exo: Bone marrow mesenchymal stem cells exosomes
CCK-8: Cell Counting kit-8
CM: Conditional media
DemiRs: Differentially expressed miRNAs
DMEM/F12: Dulbecco’s Modified Eagle Medium F12
ECAR: Extracellular acidification rate
EdU: 5-ethynyl-2’-deoxyuridine
EPCs: Endothelial progenitor cells
FBS: Fetal bovine serum
FCCP: Carbonyl cyanide 4-(trifluoromethoxy)phenylhydrazone
FIS1: Mitochondrial fission protein 1
GO: Gene Ontology
HE: Hematoxylin and eosin
HIF-1α: Hypoxia and Hypoxia-inducible factor-1 alpha
HK2: Hexokinase 2
IF: Immunofluorescent
KEGG: Kyoto Encyclopedia of Genes and Genomes
KLF5: Krüppel-like factor 5
LDH: Lactate dehydrogenase
MFN1: Mitofusin 1
MFN2: Mitofusin 2
NTA: Nanoparticle tracking analysis
OCR: Oxygen consumption rate
OVX: Ovariectomy
OXPHOS: Oxidative phosphorylation
p-Akt: phosphate-AKT
PBS: Phosphate-buffered saline
PI3k: Phosphatidylinositol 3-kinase
PKM2: Pyruvate kinase M2
PPARα: Peroxisome proliferators-activated receptors alpha
PPARγ: Peroxisome proliferators-activated receptors gamma
p-PI3k: Phosphate- phosphatidylinositol 3-kinase
qRT-PCR: Quantitative reverse transcription-polymerase chain reaction
SD: Standard deviation
SLIT3: Slit guidance ligand 3
SOX2: SRY-Box Transcription Factor 2
SPF: Specific pathogen-free
TEM: Transmission electron microscope
VASH1: Vasohibin 1
VEGF: Vascular endothelial grow factor
WCGNA: Weighted correlation network analysis
µCT: Micro computed tomography
