# Supplementary material for: Exosomes derived from BMSCs in osteogenic differentiation promote type H blood vessel angiogenesis through miR-150-5p mediated metabolic reprogramming of endothelial cells
Source: Cell Mol Life Sci. 2024 Aug 12;81(1):344. doi: 10.1007/s00018-024-05371-4 (PMC11335269; doi:10.1007/s00018-024-05371-4)
Supplement: Supplementary file 1 — Supplementary Material 1 [file 18_2024_5371_MOESM1_ESM.docx]

Additional file 1:

Additional Table 1 The list of primers.

| Gene | Forward primer (5’→3’) | Reverse primer (5’→3’) |
| --- | --- | --- |
| mmu *cd31* | CACCCATCACTTACCACCTTATG | TGTCTCTGGTGGGCTTATCT |
| mmu *emcn* | TTACTCTGGTGGGTTTGTACTG | AGCAGCTTCACACTCTCTTTAT |
| mmu *Sox2* | CATCCCAATTGCACTTCGCC | CGAGCTGGTCATGGAGTTGT |
| mmu *hif-1α* | TGAGTTCTGAACGTCGAAAAGA | GTGGCAACTGATGAGCAAGC |
| mmu *hk2* | TGATCGCCTGCTTATTCACGG | AACCGCCTAGAAATCTCCAGA |
| mmu *ldh* | TGTCTCCAGCAAAGACTACTGT | GACTGTACTTGACAATGTTGGGA |
| mmu *pkm2* | CATTACCAGCGACCCCACAG | GAGCACTCCTGCCAGACTTG |
| mmu *ppar-α* | TCGGCGAACTATTCGGCTG | GCACTTGTGAAAACGGCAGT |
| mmu *ppar-γ* | GCCCTTTGGTGACTTTATGGAG | GCAGCAGGTTGTCTTGGATG |
| mmu *β-actin* | TGTCCCTGTATGCCTCTGGT | GATGTCACGCACGATTTCC |
| mmu miR-150-5p | GCGTCTCCCAACCCTTGTA | AGTGCAGGGTCCGAGGTATT |
| mmu U6 | GCTTCGGCAGCACATATACTAAAAT | CGCTTCACGAATTTGCGTGTCAT |
