# Supplementary material for: Exosomes derived from BMSCs in osteogenic differentiation promote type H blood vessel angiogenesis through miR-150-5p mediated metabolic reprogramming of endothelial cells
Source: Cell Mol Life Sci. 2024 Aug 12;81(1):344. doi: 10.1007/s00018-024-05371-4 (PMC11335269; doi:10.1007/s00018-024-05371-4)
Supplement: Supplementary file 3 — Supplementary Material 3 [file 18_2024_5371_MOESM3_ESM.docx]

Additional file 2: **Fig. S1** Characterization of BMSCs


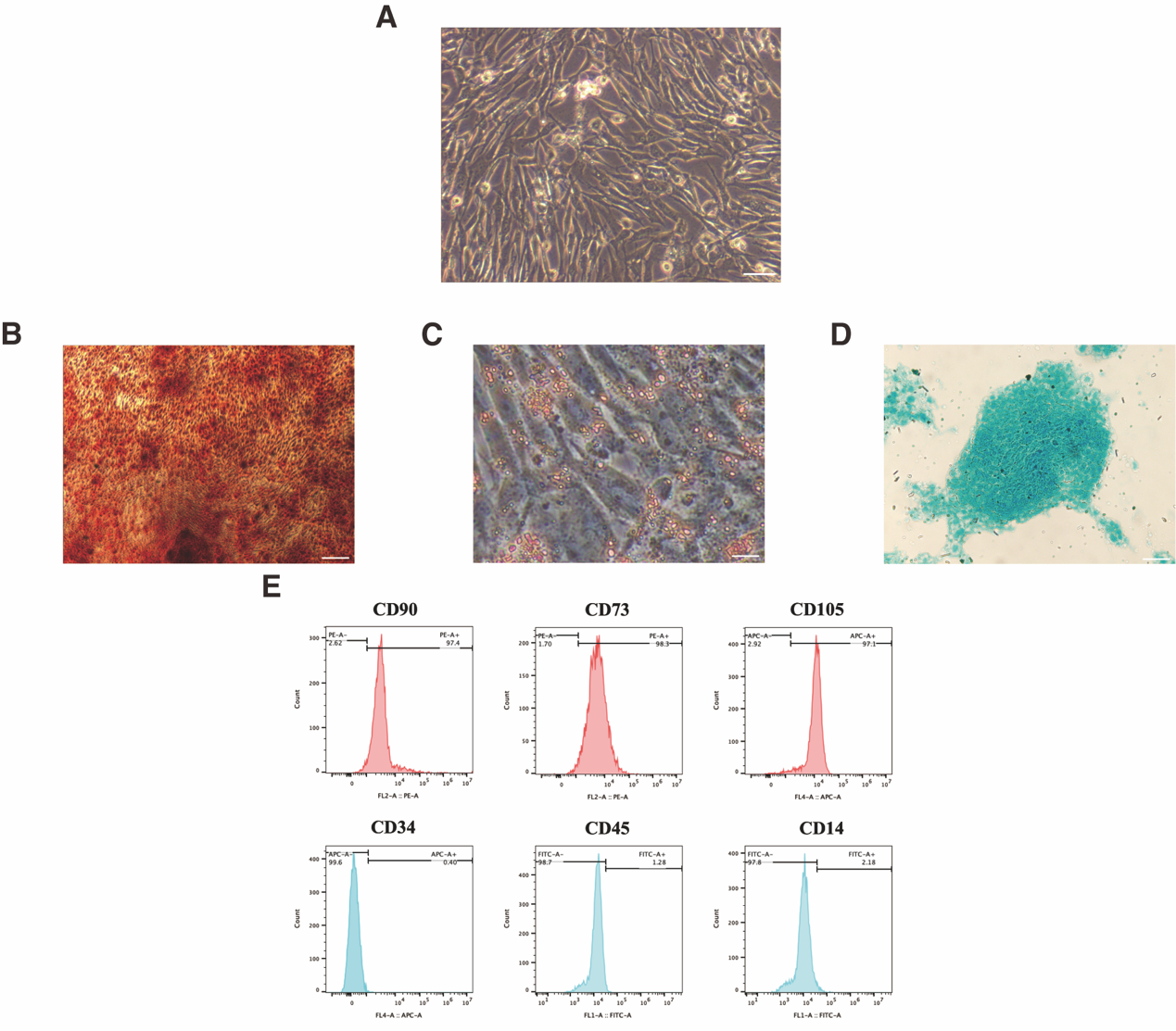


**Fig S1.** Characterization of BMSCs. **(A)** Representative images of the morphology of BSMCs. **(B-D)** Representative images of staining with alizarin red **(B)**, oil red O **(C)**, alcian blue **(D)** was performed to test the osteogenic, adipogenic, and chondrogenic differentiation of BMSCs, respectively. **(E)** Flow cytometry analysis of the phenotype profile of BMSCs (positive makers: CD90, CD73 and CD105; negative maker: CD34, CD45 and CD14). n =3. Scale bar in A=50μm, B=250μm, C=25μm, D=25μm.
