# Supplementary material for: Exosomes derived from BMSCs in osteogenic differentiation promote type H blood vessel angiogenesis through miR-150-5p mediated metabolic reprogramming of endothelial cells
Source: Cell Mol Life Sci. 2024 Aug 12;81(1):344. doi: 10.1007/s00018-024-05371-4 (PMC11335269; doi:10.1007/s00018-024-05371-4)
Supplement: Supplementary file 4 — Supplementary Material 4 [file 18_2024_5371_MOESM4_ESM.docx]

Additional file 3: **Fig. S2** Characterization of EPCs


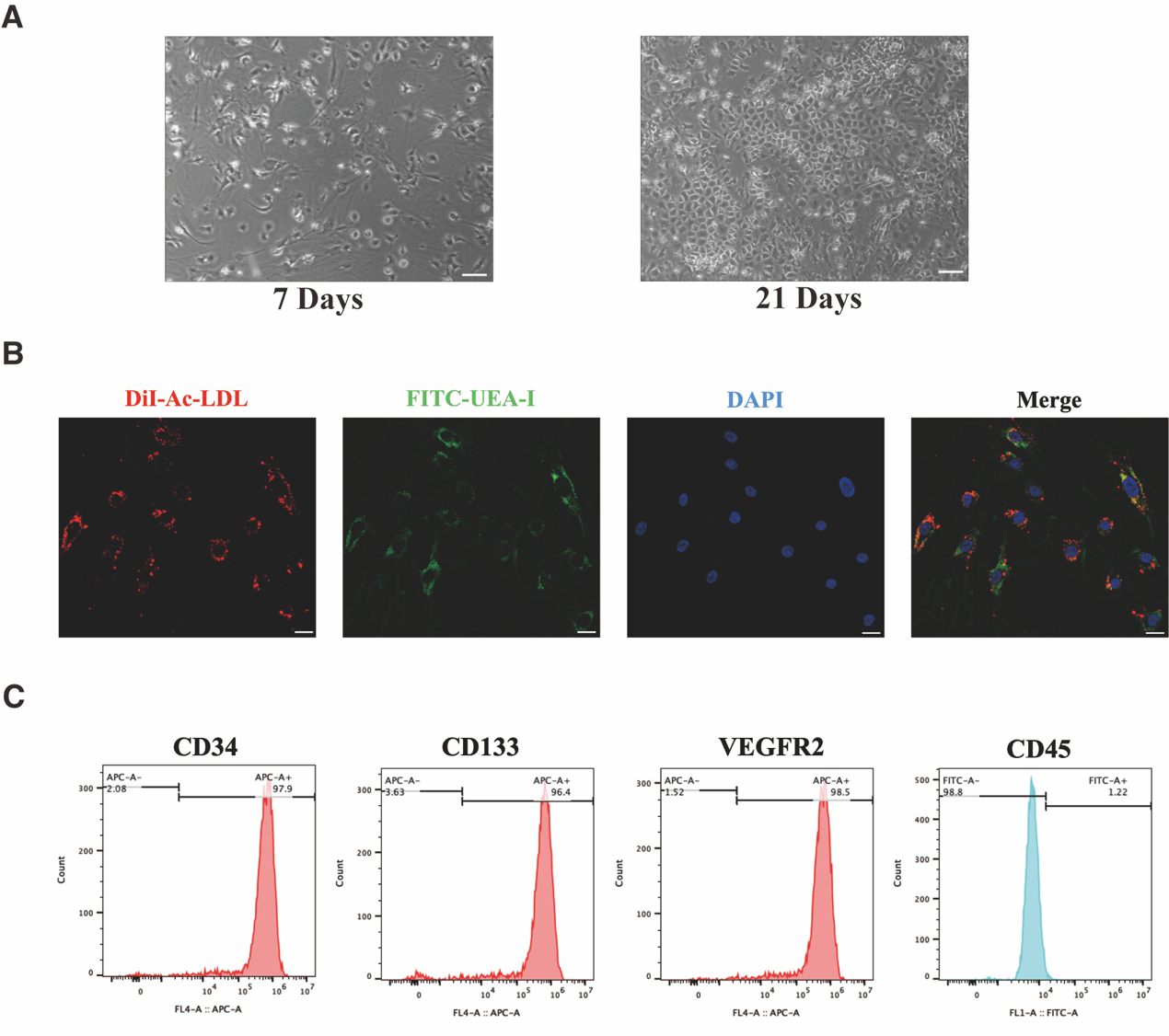


**Fig S2.** Characterization of EPCs. **(A)** Represent images of the morphology of EPCs on 7days and 21days after isolation; **(B)** Represent images of the fluorescent imaging indicating FITC-UEA-1 (green), Dil-Ac- LDL (red) and Nuclei (blue) in EPCs; **(C)** Flow cytometry analysis of the phenotype profile of EPCs (positive makers: CD34, CD133 and VEGFR2; negative maker: CD45). Scale bar in A=50μm, B=40μm.
