# Supplementary material for: Exosomes derived from BMSCs in osteogenic differentiation promote type H blood vessel angiogenesis through miR-150-5p mediated metabolic reprogramming of endothelial cells
Source: Cell Mol Life Sci. 2024 Aug 12;81(1):344. doi: 10.1007/s00018-024-05371-4 (PMC11335269; doi:10.1007/s00018-024-05371-4)
Supplement: Supplementary file 5 — Supplementary Material 5 [file 18_2024_5371_MOESM5_ESM.docx]

Additional file 4: **Fig. S3** BMSCs in osteogenic differentiation conditional medium promote ECs migration.


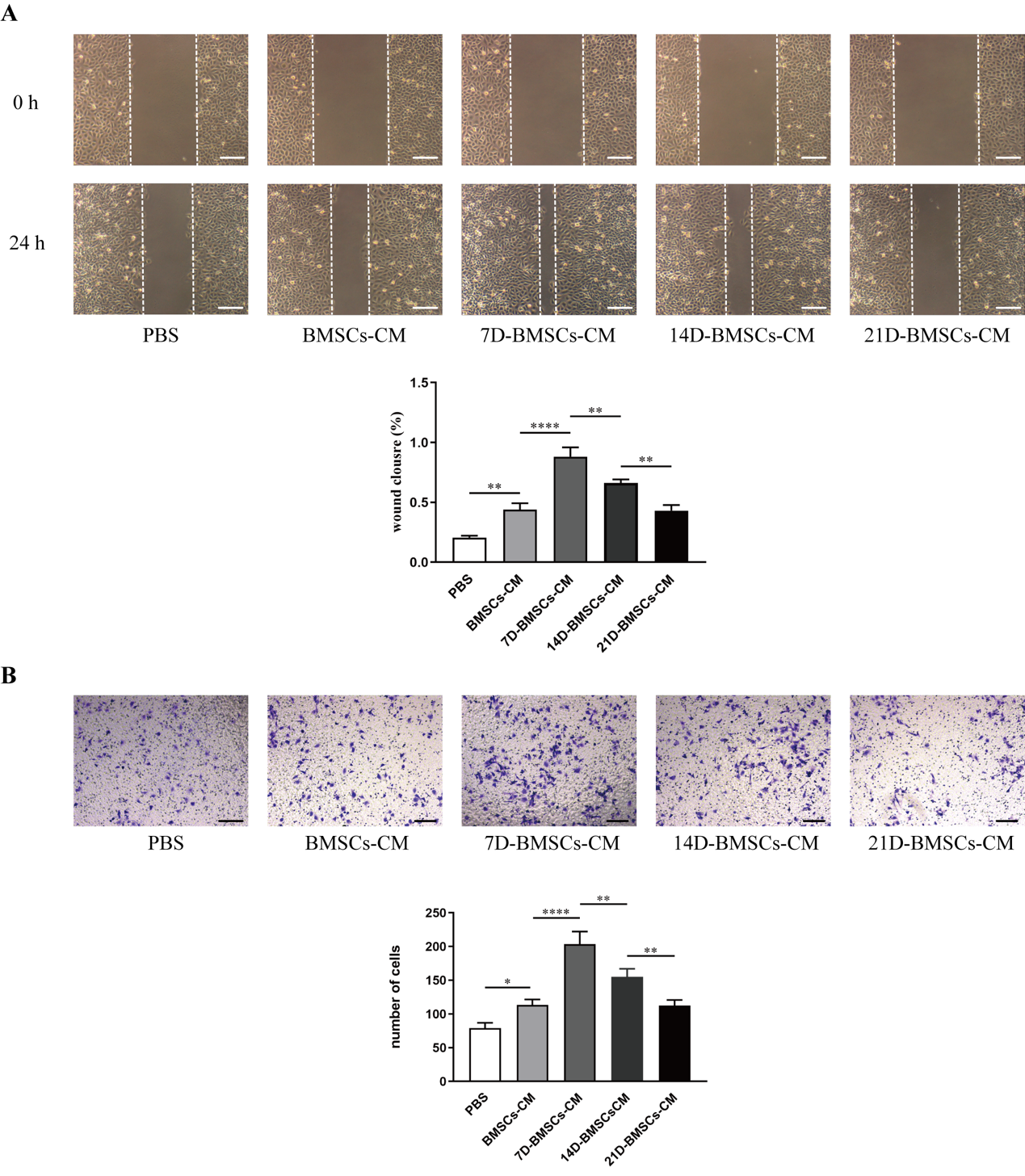


**Fig S3.** BMSCs in osteogenic differentiation conditional medium promote ECs migration. **(A)** Represent images and statistical data showed scratch wound assays of different time of osteogenic BMSCs conditional media at time 0 and 24 h, n=3. (B) Represent images and statistical data showed Transwell assays of different time of osteogenic BMSCs conditional media after 24 h, n =3. scale bar in A=100μm, B=75μm *p<0.05; **p<0.01; ***p<0.001; ****p<0.0001; ns, non-significant difference.
